# Comparative evaluation of metabolomic, microbial, and integrated features for distinguishing a rat model of heatstroke death from cervical-dislocation controls and estimating postmortem interval across organs

**DOI:** 10.3389/fmicb.2026.1897987

**Published:** 2026-09-29

**Authors:** Jianghuan Lu, Zhiao Duan, Siqi Chen, Yixin Ma, Xiaoshi Qin, Yunchao Zhou, Yuzhao Xu, Shan Ha, Jianhua Chen, Jianqiang Deng

**Affiliations:** 1 Key Laboratory of the Tropical Translational Medicine of Ministry of Education, School of Basic Medical Sciences, Hainan Academy of Medical Sciences, Hainan Medical University, Haikou, Hainan Province, China; 2 Department of Forensic Medicine & Hainan Provincial Tropical Forensic Engineering Research Center & Hainan Provincial Tropical Forensic Academician Workstation, School of Basic Medical Sciences, Hainan Medical University, Haikou, Hainan Province, China

**Keywords:** forensic microbiology, heatstroke death, machine learning, multi-omics integration, organ-specific analysis, postmortem interval, postmortem microbial profiling, untargeted metabolomics

## Abstract

**Objective:**

Postmortem diagnosis of heatstroke death and postmortem interval (PMI) estimation remain challenging in forensic practice. This study aimed to evaluate the value of untargeted metabolomics, postmortem microbial profiling, and integrated metabolite–genus features for distinguishing a rat model of heatstroke death from cervical-dislocation controls and estimating PMI across multiple organs.

**Methods:**

A controlled rat model of heatstroke death (HS) and a cervical-dislocation control group (C) were established. Brain, cerebellum, liver, kidney, and skeletal muscle samples were collected at multiple PMIs and analyzed by untargeted metabolomics and 16S rRNA sequencing. Organ-specific machine learning models were constructed using metabolomic, microbial, and integrated features. For HS/C classification, PMI-matched model-development and external-validation subsets were analyzed using random forest with repeated five-fold cross-validation, whereas PMI regression models were constructed within the HS group using XGBoost. Model performance was evaluated by cross-validation and independent external validation, and feature contribution by random forest importance and SHAP analysis.

**Results:**

Multi-organ metabolomic profiles differed between HS and C and varied with PMI, whereas postmortem microbial profiles mainly reflected organ-dependent and PMI-associated community succession. For classification, aggregated OOF AUCs ranged from 0.971 to 1.000 for metabolomics, 0.837–0.918 for microbial profiling, and 0.986–1.000 for integrated models. Integration provided limited and inconsistent numerical gains over metabolomics alone, and feature-contribution analysis indicated that the discriminative signal was dominated by metabolites. For PMI regression, liver, kidney, and skeletal muscle showed stable external validation performance across their longer available PMI ranges, whereas brain and cerebellum were evaluated over shorter sampling windows. Integrated regression models achieved external validation *R*^2^ values of 0.749–0.979, and SHAP analysis again indicated predominantly metabolomic contributions.

**Conclusion:**

Postmortem metabolomic and microbial profiles provided complementary but unequal predictive information in this controlled heatstroke model. Metabolomics was the principal information source for distinguishing HS from cervical-dislocation controls and estimating PMI, whereas microbial profiling mainly reflected PMI-associated succession. Under the present experimental setup, adding 16S-derived genus profiles provided limited additional value for classification and should be regarded mainly as a comparative or auxiliary component rather than a necessary routine layer.

## Introduction

Heatstroke is a severe heat-related condition characterized by marked hyperthermia, central nervous system dysfunction, and systemic organ injury. It may develop after passive exposure to a hot environment or during intense physical activity, and fatal cases can progress rapidly to multiorgan failure (Epstein and Yanovich, 2019; Bouchama et al., 2022). In forensic practice, the postmortem diagnosis of heatstroke death remains challenging. Scene information, environmental conditions, medical history, and exclusion of other causes of death are often essential, because autopsy and histopathological findings are usually not sufficiently specific. Recent proteomic work has explored liver proteins as candidate biomarkers for the postmortem diagnosis of heatstroke, but robust molecular evidence that can be applied under different postmortem conditions is still limited (Pan et al., 2025).

Heatstroke was selected not as a representative common cause of death, but as a diagnostically challenging forensic test case. Its growing public-health and forensic relevance in the context of increasingly frequent extreme-heat exposure further supports the need for objective molecular evidence that may complement conventional death investigation (Epstein and Yanovich, 2019; Bouchama et al., 2022; Pan et al., 2025). At the same time, terminal hyperthermia may alter early postmortem biochemical and microbial progression, making heatstroke a scientifically informative but methodologically demanding model.

Postmortem interval (PMI) estimation is another central task in death investigation. Traditional PMI assessment relies on body cooling, livor mortis, rigor mortis, decomposition, and contextual information, but these indicators are influenced by environmental conditions and individual variability. Molecular changes after death may provide additional information for PMI inference. Untargeted metabolomics can capture broad changes in small molecules and has been increasingly used to describe postmortem biochemical variation. Previous studies have shown that postmortem metabolite profiles contain time-related information and may help identify candidate molecular markers for PMI estimation (Pesko et al., 2020). Metabolomics combined with machine learning has also been applied to forensic classification and postmortem submersion interval estimation, suggesting that metabolic profiles may support both cause-of-death discrimination and temporal inference (Zhang et al., 2022). Multi-organ metabolomic studies further indicate that different tissues may preserve PMI-related information to different degrees (Lu et al., 2023).

The postmortem microbiome has also attracted increasing attention in forensic science. After death, microbial communities change with tissue degradation, nutrient redistribution, oxygen depletion, and environmental exposure. These changes are related to decomposition and may provide temporal information. Systematic reviews have suggested that 16S rRNA sequencing and microbiome profiling have potential value for PMI estimation, although their performance is affected by sampling site, environment, sequencing strategy, statistical model, and study design (Tozzo et al., 2022; Moitas et al., 2024). Human cadaver studies have shown that internal organs undergo clear microbial changes over extended postmortem intervals (Lutz et al., 2020). More recent work integrating microbial and metabolomic data has suggested that decomposition involves coordinated microbial and metabolic processes, but it also emphasizes the need for controlled study designs and independent validation before forensic application (Burcham et al., 2024).

Despite these developments, metabolomic and microbial information have rarely been evaluated together for heatstroke death investigation. Existing forensic omics studies have mainly focused on PMI estimation, single tissue types, or one omics layer. Multi-omics integration may improve model performance by combining complementary molecular features, but its added value and practical feasibility need to be evaluated carefully, especially when one omics layer already provides high predictive performance. In particular, integrated models should not be interpreted directly as mechanistic evidence, because different feature types may contribute unequally to model performance. Recent forensic multi-omics studies have demonstrated the potential of integrating metabolomics, lipidomics, proteomics, or other molecular features for PMI estimation, while also showing that organ selection, feature stability, and external validation remain important issues (Bonicelli et al., 2022; Li et al., 2024).

In this study, we established a controlled rat model of heatstroke death and a control group euthanized by cervical dislocation. Brain, cerebellum, liver, kidney, and skeletal muscle samples were collected at multiple PMIs and analyzed using untargeted metabolomics and 16S rRNA microbial profiling. We compared metabolomic features, genus-level microbial profiles, and integrated metabolite–genus features in two forensic tasks: distinguishing the present rat model of heatstroke death from controls euthanized by cervical dislocation and estimating PMI within the HS group. Model performance was evaluated across organs using cross-validation and independent external validation. Feature importance and SHAP analysis were further used to interpret the integrated models.

## Materials and methods

### Animal model and sample collection

Male Sprague–Dawley rats weighing 220–250 g were purchased from SPF Biotechnology Co., Ltd. (Beijing, China). Animals were housed under controlled conditions with free access to food and water before the experiments. To establish the heatstroke death (HS) model, rats were placed in an artificial climate chamber at 40 °C and 65% relative humidity. Rectal temperature was monitored every 10 min, and heatstroke was considered established when the rectal temperature exceeded 42 °C on three consecutive measurements. The animals were then maintained under the same heat exposure until death. Control (C) animals were euthanized by cervical dislocation at room temperature in a well-ventilated laboratory, without heat exposure. Accordingly, the HS/C comparison was confounded by terminal thermal history and agonal duration: HS animals died after sustained hyperthermia and a prolonged agonal process, whereas C animals died rapidly at room temperature. No additional control group was included to match terminal body temperature or agonal duration. After death, all carcasses were maintained at 25 °C and 65% relative humidity until sampling. All animal procedures were approved by the institutional animal ethics committee and were conducted in accordance with relevant guidelines and regulations.

Under these postmortem conditions, brain, cerebellum, liver, kidney, and skeletal muscle tissues were collected at the predefined PMI points for untargeted metabolomic analysis and 16S rRNA microbial profiling. For liver, kidney, and skeletal muscle, samples were collected at 0, 12, 24, 48, 72, 96, 120, 144, and 168 h postmortem. Brain samples were collected up to 72 h postmortem. Cerebellum samples were collected up to 48 h in the HS group and up to 72 h in the C group because of tissue decomposition and sampling limitations at later PMI points. Five biological replicates were included for each group at each PMI point.

An external validation cohort independent of the model-development cohort was established to evaluate model generalizability. These validation samples were not used for feature selection, model training, cross-validation, hyperparameter selection, or model interpretation. Because the HS/C classification and PMI regression tasks had different objectives, task-specific external validation subsets were used.

For the HS/C classification task, independent samples from the predefined HS and C experimental groups were included. For the brain, the external classification validation set contained 15 HS and 15 C samples, with three samples per group at 0, 12, 24, 48, and 72 h. For the liver, kidney, and skeletal muscle, each external classification validation set contained 27 HS and 27 C samples, with three samples per group at 0, 12, 24, 48, 72, 96, 120, 144, and 168 h. For the cerebellum, HS samples were available only up to 48 h, whereas C samples were available up to 72 h. Therefore, the external classification analysis was recalculated using a conservative matched-PMI validation subset restricted to the common PMI points of 0, 12, 24, and 48 h, with three samples per group at each time point. This resulted in 12 HS and 12 C cerebellum samples for the external confusion matrix. Unmatched cerebellar C samples at 72 h and any additional samples outside the matched-PMI structure were excluded from the matched-PMI external confusion matrix.

For the PMI regression task, external validation was performed only in HS samples because the regression models were constructed within the HS group. These independent HS validation samples were collected at random, non-fixed PMIs within the maximum available PMI range of each organ, with the exact sampling times not restricted to the predefined model-development time points. The numbers of HS samples used for PMI regression external validation were 15 for brain, 12 for cerebellum, and 27 each for liver, kidney, and skeletal muscle. For integrated models, only samples with both metabolomic and microbial profiles were included. The composition of the task-specific external-validation subsets is summarized in Supplementary Table S1.

### Untargeted metabolomic profiling and data preprocessing

Tissue samples were prepared under low-temperature conditions. For each of the five organs, 25 mg of tissue was weighed into a microcentrifuge tube containing homogenization beads, followed by the addition of 500 μL of extraction solution containing six isotope-labeled internal standards (IS1–IS6). The internal standards were succinic acid-2,2,3,3-d4, L-leucine-d3 (methyl-d3), N-benzoyl-d5-glycine, 4-aminobutyric acid-2,2,3,3,4,4-d6, N-(carboxymethyl)-N,N,N-trimethyl-d9-ammonium chloride, and nicotinamide-2,4,5,6-d4. The same internal-standard mixture was used for all five organs; IS1–IS3 were monitored in negative-ion mode, whereas IS4-IS6 were monitored in positive-ion mode. The extraction solution consisted of methanol, acetonitrile, and water at a volume ratio of 2:2:1. Samples were homogenized at 35 Hz for 4 min and sonicated in an ice-water bath for 5 min. This process was repeated three times. The extracts were incubated at −40 °C for 1 h and centrifuged at 12,000 rpm for 15 min at 4 °C. The supernatant was collected for LC–MS/MS analysis. Pooled quality control samples were prepared by mixing aliquots from all study samples and were used to monitor analytical stability during data acquisition.

Untargeted metabolomic profiling was performed using a Vanquish ultra-high-performance liquid chromatography system coupled to an Orbitrap Exploris 120 mass spectrometer. Separation was carried out on a Waters ACQUITY UPLC BEH Amide column. The mobile phase was composed of water containing 25 mmol/L ammonium acetate and 25 mmol/L ammonia solution as phase A, and acetonitrile as phase B. The autosampler temperature was set at 4 °C, and the injection volume was 2 μL. Data were acquired in both positive and negative electrospray ionization modes. The main mass spectrometry parameters were as follows: sheath gas flow rate, 50 Arb; auxiliary gas flow rate, 15 Arb; capillary temperature, 320 °C; spray voltage, +3.8 kV in positive mode and −3.4 kV in negative mode; full MS resolution, 60,000; MS/MS resolution, 15,000; and stepped normalized collision energy, 20, 30, and 40.

Raw LC–MS/MS files were converted to mzXML format using ProteoWizard. Peak processing and metabolite annotation were performed with in-house R scripts and the BiotreeDB database. Metabolite annotation confidence was categorized using an MSI-referenced four-level scheme. Level 1 annotations were assigned when the MS1 and MS2 spectra and retention time matched those of reference standards; Level 2 annotations were assigned based on matches of the MS1 and MS2 data against public databases; Level 3 annotations were assigned based on matches of the MS1 and MS2 data and predicted retention times against a theoretical database; and Level 4 denoted unknown features. No additional targeted confirmation with authentic standards was performed for the features reported in this study. Therefore, Level 2 and Level 3 metabolite names were regarded as putative annotations. The annotated abundance matrix was then used for quality filtering and normalization. Features with a relative standard deviation greater than 30% in quality control samples were removed. Features with more than 50% missing values in any single group or across all samples were also excluded to reduce the influence of sparsely detected metabolites. For the retained features, the remaining sporadic missing values were replaced by half of the minimum positive value detected for the corresponding feature. This imputation strategy was used to handle low-abundance or non-detected signals and to allow downstream statistical analysis and machine learning, but it was not intended to distinguish analytical non-detection from true biological absence. Internal-standard normalization using the six isotope-labeled standards was applied at the individual-feature level to the retained feature-area matrix before downstream statistical analysis and machine learning. Because the same tissue mass of 25 mg was used for all samples across the five organs, no additional normalization to tissue weight was performed.

### 16S rRNA sequencing and microbial profiling

Total microbial DNA was extracted from brain, cerebellum, liver, kidney, and skeletal muscle tissues using the MagBeads FastDNA Kit for Soil according to the manufacturer’s instructions. DNA quality and concentration were assessed by agarose gel electrophoresis and a NanoDrop spectrophotometer. The V3–V4 region of the bacterial 16S rRNA gene was amplified using the primers 341F and 806R. PCR products were checked by agarose gel electrophoresis and purified before library construction.

Libraries were prepared using the Illumina TruSeq Nano DNA LT Library Prep Kit. Library quality was assessed using an Agilent Bioanalyzer, and library concentration was measured with the Quant-iT PicoGreen dsDNA Assay Kit. Extraction blanks were included during DNA extraction, no-template negative controls were included during PCR amplification, and separate negative controls were included during library preparation. All controls yielded negative results. No target amplification was detected in the extraction blanks or PCR negative controls, while the library negative controls showed a Qubit measurement of 0 and no detectable amplification. These negative controls were therefore not advanced to sequencing. Qualified libraries were sequenced on the Illumina NovaSeq 6000 platform using paired-end sequencing.

Raw sequencing reads were processed in QIIME 2 (Bolyen et al., 2019). Low-quality reads were removed, paired-end reads were merged, and chimeric sequences were filtered. Amplicon sequence variants were generated after denoising using DADA2 (Callahan et al., 2016). Taxonomic annotation was performed using the SILVA database (Quast et al., 2013). Relative abundance tables were generated at the phylum and genus levels. Genus-level abundance data were used for downstream microbial community analysis and machine learning.

Alpha diversity was evaluated using Chao1, Shannon, Simpson, and Pielou_e indices. Time-related changes within the HS and C groups were assessed for each organ. Differences between HS and C at matched PMI points were also examined with multiple-comparison adjustment. Beta diversity was calculated based on Bray–Curtis distances at the genus level. Principal coordinate analysis was used to visualize community structure. PERMANOVA with 999 permutations was used to test differences in microbial community composition across PMI strata and groups. Microbial diversity analyses were performed using QIIME 2, and the corresponding statistical summaries and visualizations were generated in Python.

### Statistical analysis and differential feature summarization

The normalized metabolite abundance matrix was used to examine the overall distribution of samples and the differences between HS and C. PCA was applied as the main unsupervised analysis to visualize sample clustering across organs, groups, PMI points, and QC samples. OPLS-DA was used as a supplementary supervised analysis to further assess the separation between HS and C, and permutation testing was performed to evaluate model reliability. PCA, differential feature summarization, and visualization of metabolomic overview results were performed in R. OPLS-DA and permutation tests were performed in SIMCA.

Differential metabolic features were screened within each organ by comparing HS and C samples at the same PMI point. Fold change and statistical significance were calculated for each feature. Features with *p* < 0.05 and |log₂ fold change| > 1 were defined as differential metabolic features. The numbers of total, increased, and decreased features were then summarized for each organ and PMI point. These counts were used to describe how the metabolic differences between HS and C changed across postmortem intervals.

In this study, differential feature counts were used to describe organ- and PMI-dependent variation under the present experimental conditions, not to infer specific metabolic or microbial mechanisms. Because missing-value imputation may affect the apparent magnitude of metabolite changes, these differential feature summaries were interpreted as descriptive patterns rather than definitive evidence of metabolite appearance or disappearance during decomposition.

### Machine learning model construction and evaluation

Machine learning models were constructed separately for each organ. Two prediction tasks were considered: classification to distinguish the present rat model of heatstroke death from controls euthanized by cervical dislocation, and PMI regression within the HS group. Three feature strategies were compared in both tasks: metabolomic features, genus-level microbial profiles, and integrated metabolite–genus features. Using the task-specific cohorts described above, feature selection and model training were confined to the development cohort, and external-validation samples were used only for final testing. For the HS/C classification task, the model-development data were restricted to PMI points represented in both groups, and equal numbers of HS and C samples were retained within each PMI stratum. The final classification development sets comprised 48 brain samples (24 HS and 24 C), 40 cerebellum samples (20 HS and 20 C), and 90 samples for each of the liver, kidney, and skeletal muscle (45 HS and 45 C per organ). In the brain, five samples per group were included at 0, 12, 24, and 48 h, and four samples per group were included at 72 h. In the cerebellum, only the common PMI points of 0, 12, 24, and 48 h were retained, and the unmatched C samples at 72 h were excluded. Only samples with paired metabolomic and microbial profiles were retained, and the same PMI-matched sample set was used for the metabolomics-based, microbial profiling-based, and integrated classification models. All machine learning analyses were implemented in Python using scikit-learn and XGBoost.

Random forest (RF) was used as the common classifier for HS/C classification across the three feature strategies. Before RF-based feature ranking and selection, the candidate feature pools contained 2,372, 2,247, 3,032, 2,992, and 3,051 metabolic features and 177, 163, 175, 230, and 165 genus-level microbial features for the brain, cerebellum, liver, kidney, and skeletal muscle, respectively. In the single-omics analyses, candidate features were ranked according to RF importance. The metabolomics-based models used the 10 highest-ranked metabolic features from each organ, whereas the microbial profiling-based models used the 10 highest-ranked genus-level microbial features from each organ. Classification models were evaluated using repeated five-fold cross-validation with five repeats. Fold assignment was jointly by HS/C group and PMI stratum, and scaling, RF-based feature ranking, and selection of the 10 highest-ranked features were performed separately for the metabolomic and microbial data within each training fold; the held-out fold was used only for prediction. The same fold assignments were reused for the metabolomics-based, microbial profiling-based, and integrated models. For each integrated-model fold, the 10 metabolic features and 10 microbial genera selected independently within that training fold were concatenated to form a 20-feature input matrix, and a new RF classifier was fitted using only the corresponding training-fold data. For external validation, scaling parameters, RF-based feature rankings, and feature selection were refitted separately for the metabolomic and microbial data using the complete model-development cohort. The final 10 metabolic features and 10 microbial genera were then combined to train the integrated RF model in the complete model-development cohort, and the fitted models and selected feature sets were applied unchanged to the independent external-validation samples. The candidate feature-pool sizes and selected input-feature numbers used for HS/C classification are summarized in Supplementary Table S2.

Classification performance was evaluated in the PMI-matched model-development cohort using the repeated five-fold cross-validation procedure described above and was subsequently tested in the independent PMI-matched external-validation cohort. Accuracy, precision, recall (sensitivity), specificity, F1-score, and the area under the receiver operating characteristic curve (AUC) were calculated, with HS treated as the positive class. Within each repeat, every sample was included once in a held-out fold and received one out-of-fold (OOF) predicted probability. The five OOF probabilities obtained for each sample across the five repeats were averaged at the sample level before calculating the aggregated OOF ROC curves and AUC values shown in Figures 1A–C. Fold-level accuracy, precision, recall, F1-score, and AUC were summarized as mean ± SD across the 25 held-out validation folds and were reported separately from the aggregated sample-level OOF AUCs. Final class labels in the external-validation cohort were assigned using a predicted-probability threshold of 0.5, and organ-level performance was summarized using confusion matrices generated from the organ-specific external-classification subsets described above. In the cerebellum, all reported external classification confusion matrices were based on the matched-PMI subset restricted to 0, 12, 24, and 48 h, with 12 HS and 12 C samples. In addition to overall organ-level performance, aggregated OOF predictions and final external-validation predictions were stratified by PMI point. Within each PMI stratum, sample counts, confusion-matrix components, accuracy, precision, recall, specificity, F1-score, and AUC were calculated. Because each PMI stratum in the external-validation cohort contained only three HS and three C samples, the PMI-specific external-validation metrics were interpreted descriptively.

**Figure 1:**
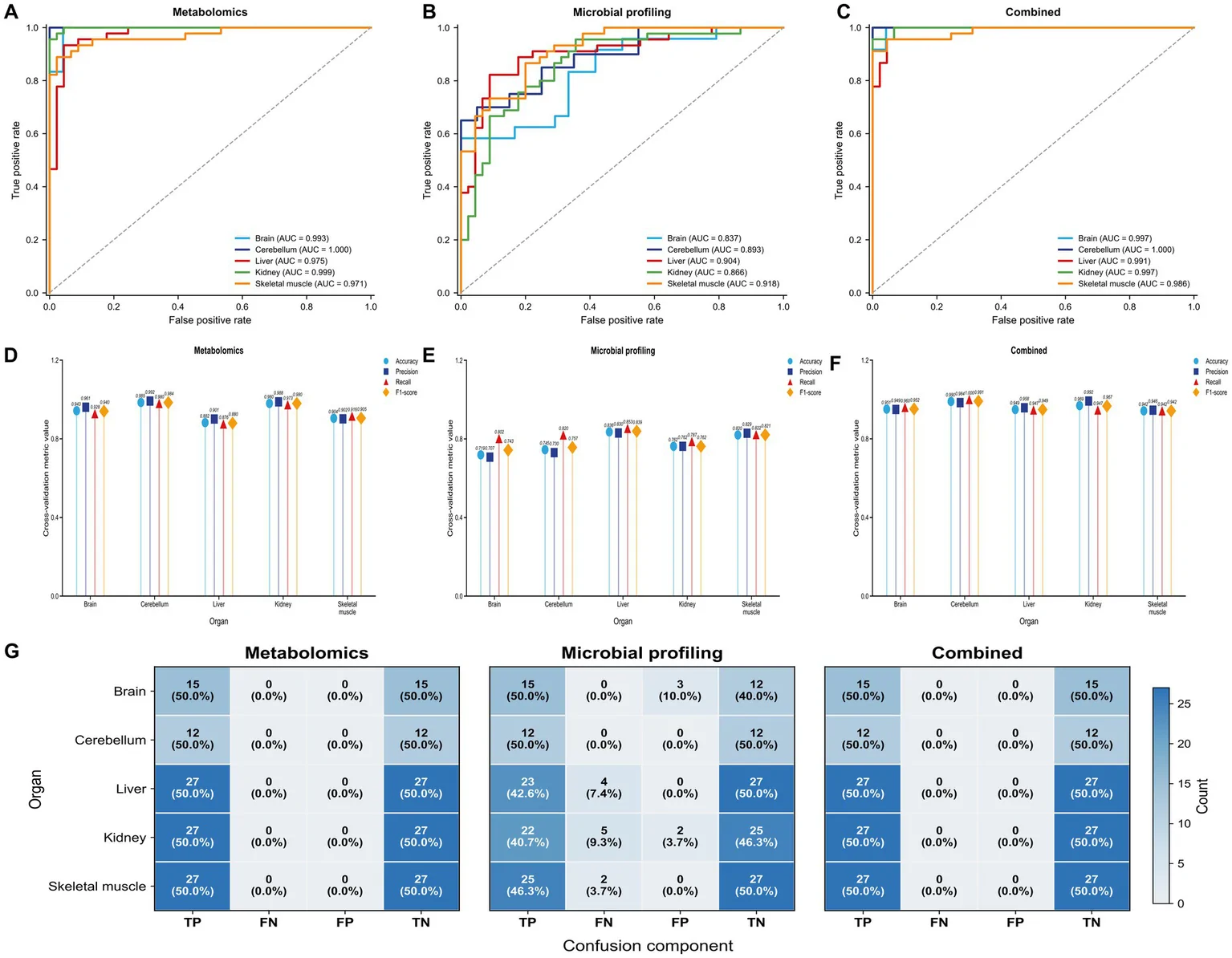
Cross-organ comparison of model performance for discriminating HS from C using metabolomic, microbial, and integrated feature sets. All classification analyses were based on PMI-matched HS/C datasets. **(A–C)** Aggregated out-of-fold ROC curves from repeated cross-validation for classification models distinguishing HS from C based on metabolomic features **(A)**, genus-level microbial profiles **(B)**, and integrated metabolite–genus features **(C)** across the five organs. **(D–F)** Fold-level cross-validation performance metrics shown as mean ± SD across the 25 held-out validation folds, including accuracy, precision, recall, and F1-score, for metabolomic models **(D)**, microbial profiling models **(E)**, and integrated models **(F)**. **(G)** External validation confusion component heatmaps for the three feature strategies across organs. HS was treated as the positive class. TP, true positive; FN, false negative; FP, false positive; TN, true negative.

PMI regression models were constructed only within the HS group. For each organ, regression models were built using metabolomic features, microbial genus profiles, and integrated metabolite–genus features. XGBoost was used for PMI regression because it can model nonlinear relationships and handle high-dimensional feature sets. Feature selection was performed within the model development cohort, and the selected features were fixed before external validation. The integrated regression models were constructed by combining the selected metabolite and microbial features from the same organ. Regression performance was assessed by comparing predicted PMI values with measured PMI values. In the main analysis, external validation performance was summarized using the coefficient of determination and mean absolute error. These metrics were used to compare the three feature strategies within each organ and to summarize model performance across organs. Because *R*^2^ is influenced by the range and distribution of the target variable, comparisons across organs with different available PMI windows were interpreted cautiously. The external validation cohort was not used during feature selection or model training. Thus, the PMI regression validation subset differed from the balanced HS/C classification validation subset and consisted only of independent HS samples collected at random, non-fixed PMIs within the available organ-specific PMI ranges.

Feature contribution analysis was performed to interpret the integrated models. For each final integrated classification model, RF importance was calculated for all 20 input features. To provide a consistent organ-level display in Figure 2A, the two metabolites with the highest RF importance and the microbial genus with the highest RF importance were selected separately for each organ. The relative contributions shown in Figure 2B were calculated by separately summing the RF importance values of all 10 metabolic features and all 10 microbial genera in each final integrated model and expressing each modality-specific sum as a percentage of the total importance. Feature directionality in Figure 2C was evaluated for the same representative features shown in Figure 2A. For each feature, log_2_(HS/C) was calculated from the unscaled abundance values in the PMI-matched model-development cohort as log_2_[(mean abundance in HS + 1 × 10^−6^)/(mean abundance in C + 1 × 10^−6^)]. These values were used only to describe group-level enrichment tendencies and were not used for model fitting or interpreted as evidence of biological causality. For the integrated PMI regression models, SHAP analysis was applied to the final XGBoost models using the SHAP package. Mean absolute SHAP values were used to identify the leading features and to calculate the relative contribution of metabolites and microbial genera. Model performance plots and feature contribution figures were generated in Python.

**Figure 2:**
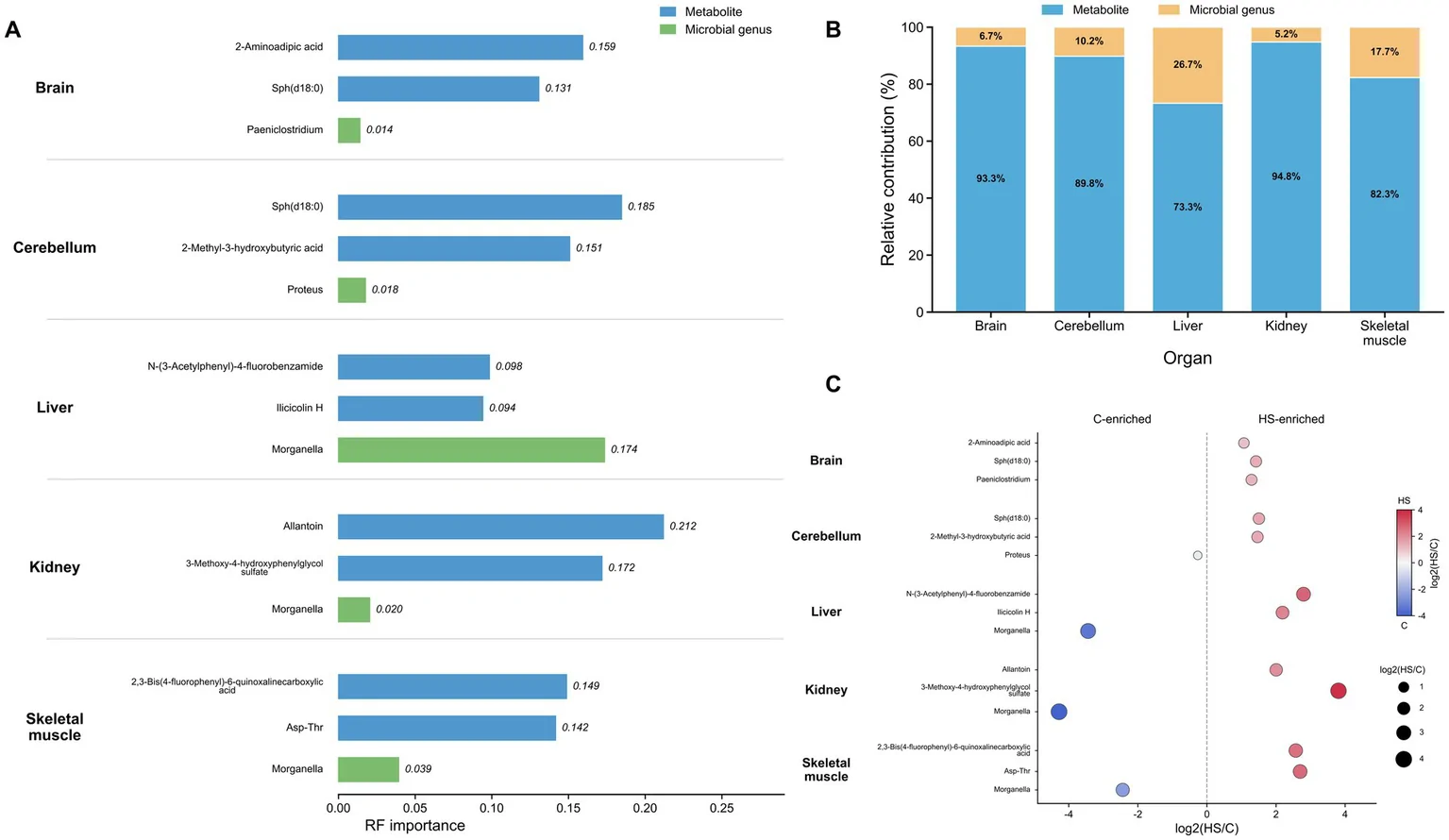
Organ-specific feature contribution and directionality in integrated models for discriminating HS from C. **(A)** For each organ, the two metabolites with the highest RF importance and the microbial genus with the highest RF importance in the final integrated classification model. **(B)** Relative contribution of all 10 selected metabolic features and all 10 selected genus-level microbial features in each integrated classification model. Blue bars indicate metabolites, and green bars indicate microbial genera. **(C)** Directionality of the same representative features shown in panel A, expressed as log2(HS/C). Bubble color represents enrichment tendency, and bubble size represents absolute magnitude of log2(HS/C). HS, heatstroke death group; C, control group; RF, random forest.

## Results

### Multi-organ untargeted metabolomic profiling and differential feature analysis

Untargeted metabolomic profiling was performed on brain, cerebellum, liver, kidney, and skeletal muscle samples. After quality control and metabolite annotation, 2,372, 2,247, 3,032, 2,992, and 3,051 MS2-annotated metabolic features were obtained from the brain, cerebellum, liver, kidney, and skeletal muscle, respectively (Figure 3F). Quality control samples clustered closely in the multivariate score plots, supporting the analytical stability of the metabolomic data. PCA showed that HS and C samples were separated to different extents across the five organs, while samples collected at different PMIs also showed shifts in the score space. OPLS-DA score plots and permutation validation results are shown in Supplementary Figures S1, S2. These results indicate that, under the present experimental conditions, multi-organ metabolic profiles differed between HS and C and were also affected by postmortem interval, with clear organ-dependent variation in the overall distribution patterns (Figure 3).

**Figure 3:**
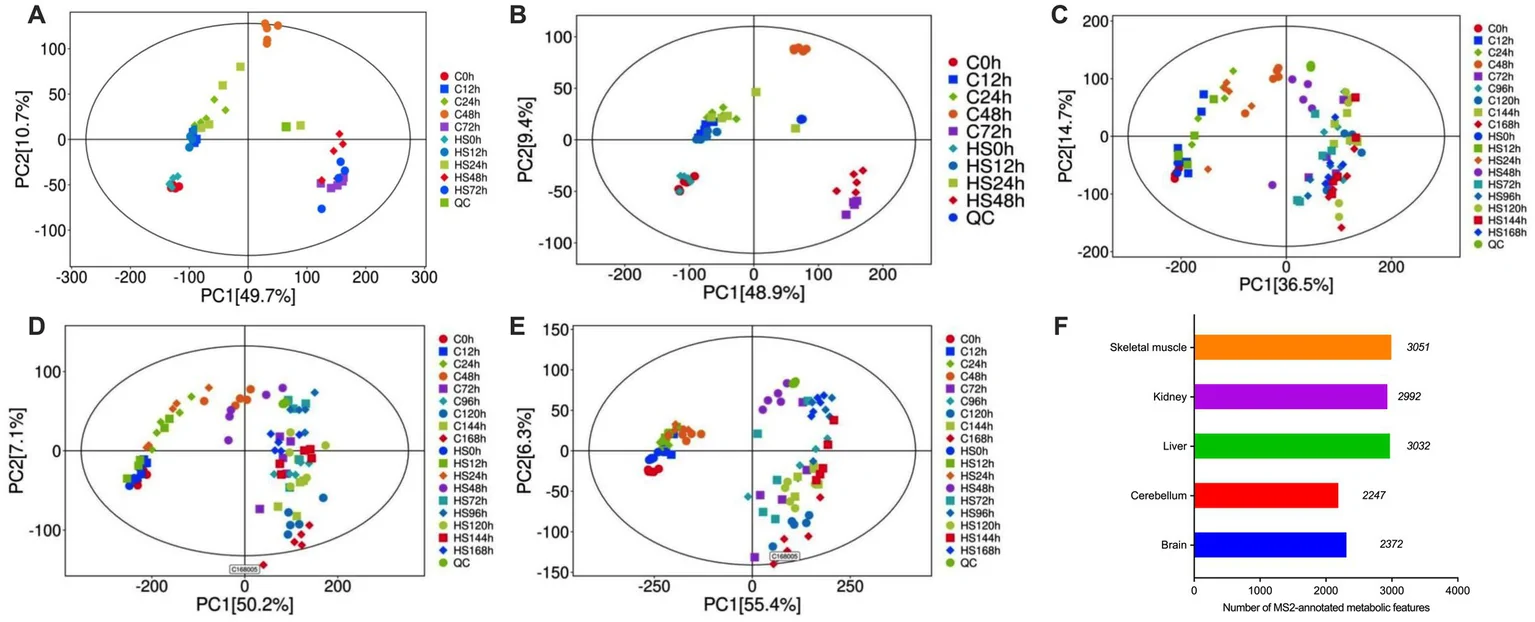
Overview of multi-organ metabolomic profiles and annotated metabolic features. **(A–E)** Principal component analysis (PCA) score plots of untargeted metabolomic profiles in the brain, cerebellum, liver, kidney, and skeletal muscle samples. The PCA plots were used to visualize the overall distribution of HS, C, and QC samples across organs. **(F)** Number of MS2-annotated metabolic features detected in each organ after quality control and annotation. HS, heatstroke death group; C, control group; QC, quality control; PMI, postmortem interval.

Differential analysis was further performed between HS and C samples at matched PMI points within each organ. The numbers of increased and decreased metabolic features varied across organs and PMI points. In the brain and cerebellum, the most prominent changes were observed at 48 h, with a larger number of increased features in HS relative to C. In the liver, kidney, and skeletal muscle, which covered the longer 0–168 h PMI range, the direction and magnitude of differential features changed over time. Some PMI points were dominated by increased features, whereas others showed a higher proportion of decreased features. These results indicate that the metabolic differences between HS and C were not fixed across the postmortem period, but showed organ- and PMI-dependent variation (Figure 4).

**Figure 4:**
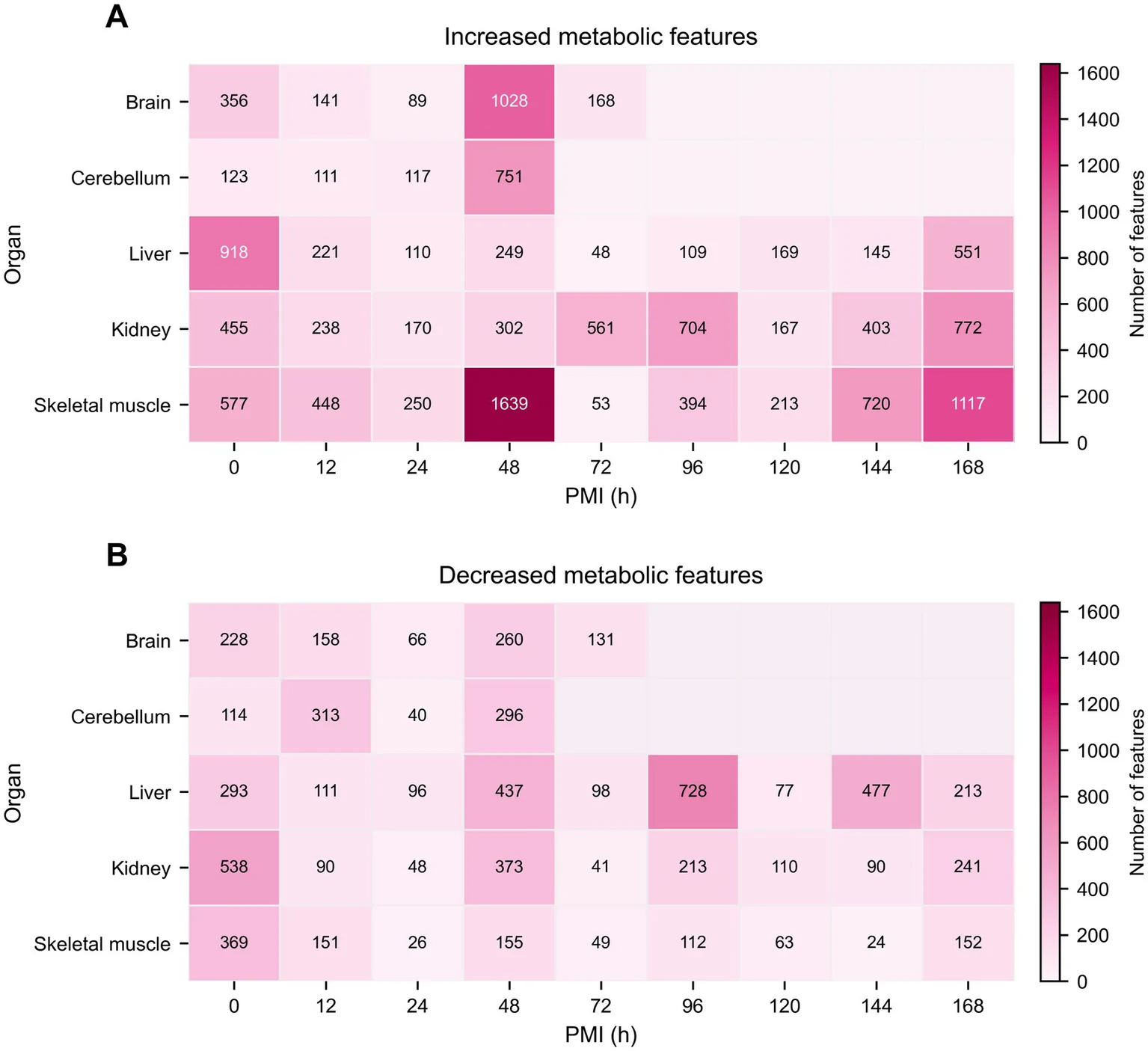
PMI-dependent variation in increased and decreased metabolic features between HS and C across organs. **(A,B)** Heatmaps showing the numbers of increased **(A)** and decreased **(B)** metabolic features in HS relative to C at matched PMI points across the brain, cerebellum, liver, kidney, and skeletal muscle. Cells without numbers indicate PMI points that were not available for the corresponding organ or comparison. HS, heatstroke death group; C, control group; PMI, postmortem interval.

Accordingly, the metabolomic data were used mainly as a quantitative basis for subsequent HS/C classification and HS-group PMI regression, rather than for direct mechanistic interpretation of heatstroke-related pathways.

### Postmortem microbial community composition and diversity analysis across organs

16S rRNA sequencing was performed on brain, cerebellum, liver, kidney, and skeletal muscle samples to characterize postmortem microbial profiles across organs. After quality control, all samples showed Good’s coverage values greater than 0.998, indicating sufficient sequencing depth for downstream community analysis. At the genus level, 177, 163, 175, 230, and 165 features were obtained from the brain, cerebellum, liver, kidney, and skeletal muscle, respectively. These genus-level profiles were then used to summarize microbial composition, diversity patterns, and PMI-associated community variation across organs.

The genus-level abundance heatmap showed that the dominant genera varied across organs and postmortem intervals. Paeniclostridium, Proteus, Hathewaya, and Morganella were repeatedly observed among the major genera, but their relative abundances differed among the five organs and changed with PMI. In the brain and cerebellum, several early and intermediate PMI points showed visible changes in Proteus, Paeniclostridium, Morganella, and Escherichia-Shigella. In the liver, kidney, and skeletal muscle, changes in Proteus and Paeniclostridium were more evident across the extended 0–168 h PMI range, with additional variation in genera such as Hathewaya and Morganella at specific time points. Overall, the genus-level profiles showed organ-dependent microbial redistribution during the postmortem period, rather than a uniform pattern across all organs (Figure 5A).

**Figure 5:**
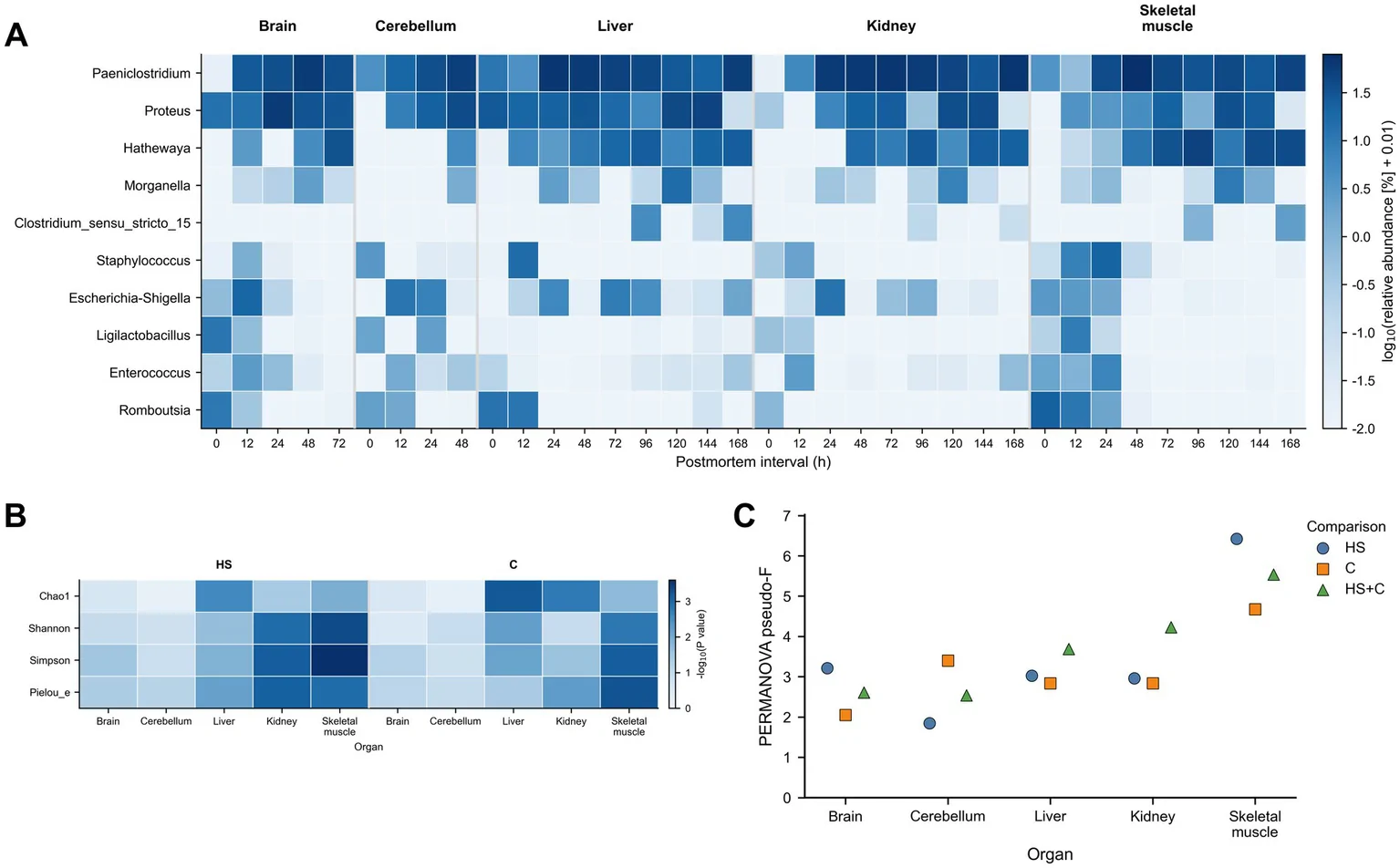
Postmortem microbial profiles across organs mainly reflected PMI-associated community succession. **(A)** Genus-level relative abundance heatmap showing the major microbial genera across brain, cerebellum, liver, kidney, and skeletal muscle samples. **(B)** Heatmap summarizing PMI-related changes in alpha diversity indices across organs and groups. **(C)** PERMANOVA summary based on Bray–Curtis distances, showing differences in microbial community structure across PMI strata within each organ. HS, heatstroke death group; C, control group; PMI, postmortem interval; PERMANOVA, permutational multivariate analysis of variance.

Alpha diversity analysis further indicated that the microbial community response to PMI differed by organ. In the HS group, liver, kidney, and skeletal muscle showed significant PMI-related changes in multiple alpha diversity indices, including Chao1, Shannon, Simpson, and Pielou_e. Similar time-related changes were also observed in the corresponding C samples. In contrast, most alpha diversity indices in the brain and cerebellum showed weaker PMI-related variation. When HS and C were compared at matched PMI points, significant differences were not consistently observed after multiple-comparison correction, except for the Shannon index in the liver at 24 and 168 h. These results suggest that alpha diversity mainly reflected time-related community changes, with limited evidence for sustained separation between HS and C at this level (Figure 5B). The detailed statistical summary of alpha diversity comparisons is provided in Supplementary Table S3.

PERMANOVA based on Bray–Curtis distances confirmed that community structure varied with PMI in all five organs. Within the HS group, the pseudo-*F* values were 3.214 for brain, 1.844 for cerebellum, 3.023 for liver, 2.961 for kidney, and 6.424 for skeletal muscle. Corresponding time-related effects were also observed within the C group, and the stratified HS + C comparison remained significant across all organs. Among the five organs, skeletal muscle showed the highest pseudo-*F* value in the HS group and in the stratified HS + C comparison, indicating a stronger PMI-associated community shift under the present experimental conditions (Figure 5C). Beta-diversity ordination plots are shown in Supplementary Figure S3, and the detailed PERMANOVA results based on Bray–Curtis distances are provided in Supplementary Table S4.

Taken together, the microbial profiles primarily reflected organ-dependent, PMI-associated succession and were therefore treated as an auxiliary source for subsequent model comparison rather than as primary evidence of stable HS/C separation.

### Comparison of HS and C classification models based on metabolomic, microbial, and integrated features

Classification performance across the five organs was compared among models based on metabolomic features, genus-level microbial profiles, and integrated metabolite-genus features (Figure 1). All analyses used PMI-matched HS/C datasets; cerebellar analyses were restricted to 0, 12, 24, and 48 h, and brain sample numbers were balanced between HS and C within each included PMI stratum.

Using metabolomic features alone, the classifiers achieved aggregated OOF AUCs of 0.993 in the brain, 1.000 in the cerebellum, 0.975 in the liver, 0.999 in the kidney, and 0.971 in skeletal muscle, with values ranging from 0.971 to 1.000 across organs (Figure 1A). All samples in the five organ-specific external-validation cohorts were correctly classified (Figure 1G).

In contrast, classifiers using only genus-level microbial profiles showed aggregated OOF AUCs of 0.837 in the brain, 0.893 in the cerebellum, 0.904 in the liver, 0.866 in the kidney, and 0.918 in skeletal muscle, corresponding to a range of 0.837–0.918 (Figure 1B). External validation showed no classification errors for the cerebellum model. The brain model produced three false positives, the liver model produced four false negatives, the kidney model produced five false negatives and two false positives, and the skeletal muscle model produced two false negatives (Figure 1G).

When the two feature types were combined, the integrated models achieved aggregated OOF AUCs of 0.997 in the brain, 1.000 in the cerebellum, 0.991 in the liver, 0.997 in the kidney, and 0.986 in skeletal muscle, with an overall range of 0.986–1.000 (Figure 1C). As observed for the metabolomic models, all samples in the five external-validation cohorts were correctly classified (Figure 1G).

Fold-level accuracy, precision, recall, and F1-score are shown in Figures 1D–F and detailed in Supplementary Table S5, while organ-level external-validation confusion-matrix metrics are provided in Supplementary Table S6. When classification performance was examined separately by PMI, the metabolomic and integrated models generally retained high discrimination across the available postmortem intervals, including 0, 72, and 168 h where available, whereas the microbial-only models showed greater variation among PMI strata. In the aggregated OOF analysis, reduced microbial-model performance was observed, for example, in the brain at 72 h (accuracy, 0.375; AUC, 0.750) and in the kidney at 0 h (accuracy, 0.500; AUC, 0.400). In external validation, the metabolomic and integrated models correctly classified all samples at every available PMI point, whereas errors in the microbial models were confined to selected PMI strata. Complete PMI-stratified aggregated OOF and external-validation metrics are provided in Supplementary Tables S7, S8, respectively. Because each PMI-specific external-validation subset contained only three HS and three C samples, these results were interpreted descriptively.

### Feature importance and directionality analysis of integrated classification models

To further interpret the integrated models for distinguishing HS from C, feature contribution and directionality were examined across the five organs. In each organ, the representative features displayed in Figure 2A comprised the two metabolites with the highest RF importance and the microbial genus with the highest RF importance. The representative features differed among organs, indicating that the integrated discrimination models relied on organ-specific feature combinations rather than a uniform feature set shared by all tissues.

The relative contribution analysis further showed that metabolites contributed the majority of the model importance in all five organs, whereas microbial genera accounted for a smaller proportion. This pattern was consistent across brain, cerebellum, liver, kidney, and skeletal muscle, supporting that the main discriminative signal in the integrated models was derived from metabolomic features. Microbial genera provided additional information, but their contribution was limited compared with that of metabolites (Figure 2B).

The directionality analysis based on log2(HS/C) showed that the representative features displayed in Figure 2A exhibited different enrichment tendencies between HS and C. Some features were relatively enriched in HS, whereas others were relatively enriched in C, and these patterns varied across organs. The directionality results were used only to describe feature-level differences between HS and C in the classification task and were not interpreted as direct evidence of heatstroke-specific metabolic or microbial mechanisms (Figure 2C). The complete list of selected features, RF importance values, and log2(HS/C) values is provided in Supplementary Table S9.

Overall, the integrated classification models showed that microbial genera could supplement the feature space for distinguishing HS from C, but the discrimination was predominantly driven by metabolites. Together with the model performance comparison, these findings indicate that adding microbial features produced limited additional gain over metabolomics alone in the classification task. Therefore, the integrated strategy should mainly be viewed as a comparative approach for evaluating omics-layer contributions, rather than as evidence that multi-omics integration is necessary for classification under the present experimental setup.

### Comparison of PMI regression models based on metabolomic, microbial, and integrated features

PMI regression models were constructed within the HS group using metabolomic features, microbial genus profiles, and integrated metabolite–genus features across the five organs. Model performance was mainly evaluated using external validation *R*^2^ and MAE. Across the three feature strategies, external validation results differed markedly among organs and available PMI ranges. Liver, kidney, and skeletal muscle showed stable prediction performance within the longer 0–168 h sampling window, whereas brain and cerebellum were evaluated over shorter PMI windows because of tissue decomposition and sampling limitations (Figures 6A–D).

**Figure 6:**
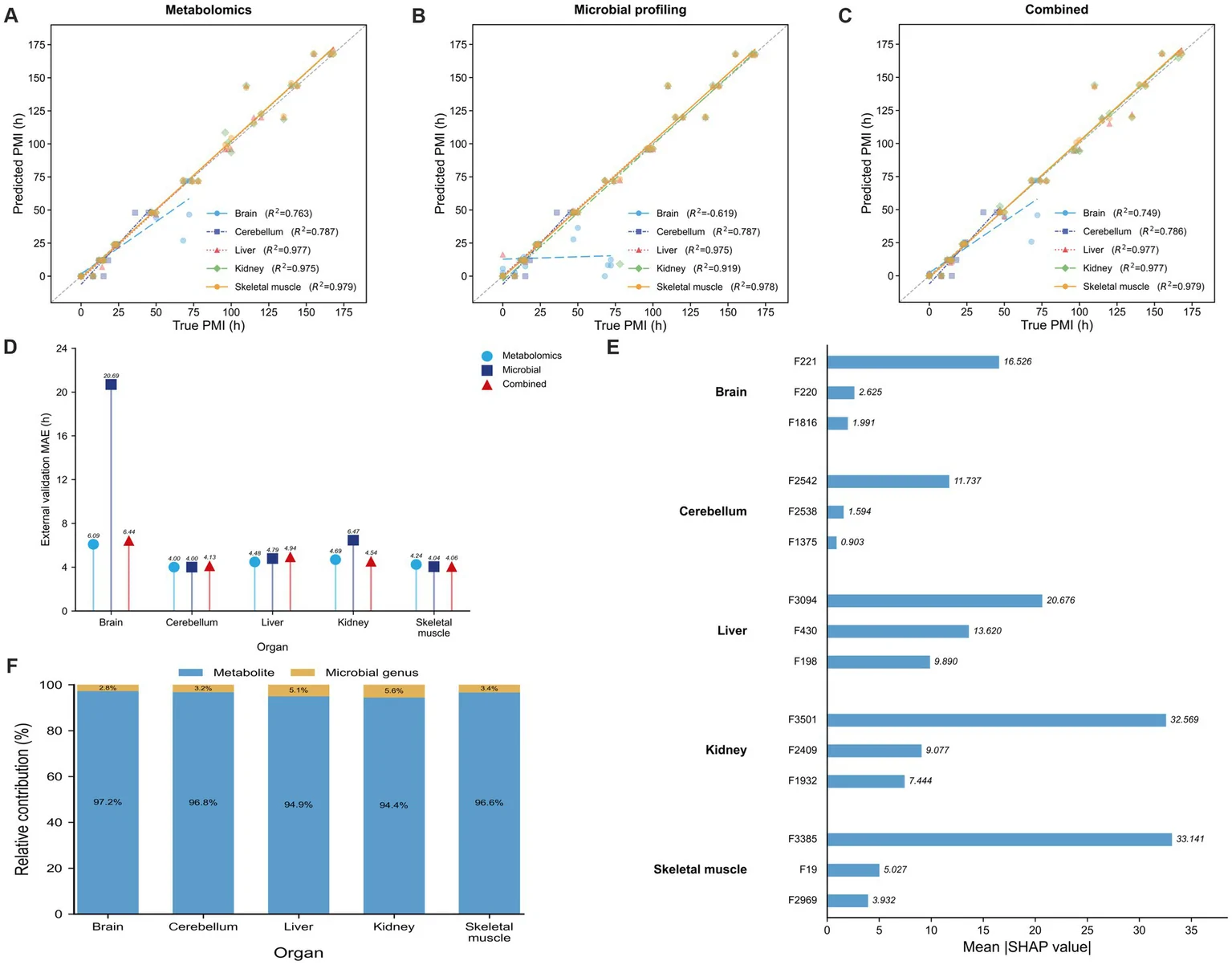
External validation performance and feature contribution of PMI regression models across organs. **(A–C)** External validation *R*^2^ values of PMI regression models based on metabolomic features **(A)**, genus-level microbial profiles **(B)**, and integrated metabolite–genus features **(C)** across the five organs. **(D)** External validation MAE values of the three feature strategies across organs. **(E)** The three features with the highest mean absolute SHAP values for each organ in the integrated PMI regression models. All features displayed in panel E were metabolic features and are labeled using organ-specific feature IDs; the corresponding database annotations and annotation-confidence information are provided in Supplementary Table S12. **(F)** Relative contribution of metabolites and microbial genera in the integrated PMI regression models. PMI, postmortem interval; MAE, mean absolute error; *R*^2^, coefficient of determination; SHAP, Shapley additive explanations.

For the metabolomics-based models, external validation *R*^2^ values ranged from 0.763 to 0.979 (Figure 6A), with MAE values ranging from 4.00 to 6.09 h (Figure 6D). Skeletal muscle, liver, and kidney showed higher *R*^2^ values of 0.979, 0.977, and 0.975, respectively, whereas brain and cerebellum showed lower *R*^2^ values of 0.763 and 0.787. These results indicate that metabolomic features provided usable PMI-related information in all five organs, but the higher *R*^2^ values observed in liver, kidney, and skeletal muscle should be interpreted in the context of their broader PMI range.

Microbial profiling-based models showed greater organ-dependent variability. External validation *R*^2^ values ranged from −0.619 to 0.978 (Figure 6B). Skeletal muscle and liver retained high *R*^2^ values of 0.978 and 0.975, respectively, whereas the brain model showed poor external validation performance, with an *R*^2^ of −0.619. The corresponding MAE values also showed wider variation, with the brain reaching 20.69 h (Figure 6D). Compared with metabolomics-based models, microbial profiling showed less consistent PMI regression performance across organs.

The integrated metabolite–genus models achieved external validation *R*^2^ values ranging from 0.749 to 0.979 (Figure 6C) and MAE values ranging from 4.06 to 6.44 h (Figure 6D). In liver, kidney, and skeletal muscle, integrated models maintained stable external validation performance within the 0–168 h sampling window, with *R*^2^ values of 0.977, 0.977, and 0.979, respectively. In brain and cerebellum, the integrated models showed *R*^2^ values of 0.749 and 0.786, respectively, which were close to or slightly lower than the corresponding metabolomics-based models. Overall, integrated modeling improved prediction performance relative to microbial profiling in most organs, while showing essentially comparable performance in the cerebellum, and did not consistently outperform metabolomics-based modeling across all organs.

Taken together, PMI regression performance was strongly dependent on both organ type and available PMI range under the present experimental conditions. Liver, kidney, and skeletal muscle showed stable external validation results across the three feature strategies within the 0–168 h sampling window, whereas brain and cerebellum were evaluated over shorter windows and therefore should not be directly compared with peripheral organs solely on the basis of *R*^2^. These results indicate that organ selection and PMI coverage both affect PMI regression performance. Because all regression models were developed and externally validated exclusively within HS animals, these performance estimates describe PMI prediction within the present HS model rather than a cause-independent PMI model. Detailed cross-validation and external validation metrics for all PMI regression models are provided in Supplementary Table S10.

### SHAP-based feature contribution analysis of integrated PMI regression models

To further interpret the integrated PMI regression models, the three features with the highest mean absolute SHAP values were summarized for each organ. All 15 features displayed in Figure 6E were metabolic features, although the specific features differed among organs. In the brain, F221, F220, and F1816 showed the highest mean absolute SHAP values; in the cerebellum, F2542, F2538, and F1375; in the liver, F3094, F430, and F198; in the kidney, F3501, F2409, and F1932; and in skeletal muscle, F3385, F19, and F2969. Among these features, F221, F220, F430, F198, and F19 were assigned Level 1 annotations, whereas the other ten features were assigned Level 2 or Level 3 annotations and were therefore treated as putative annotations. Complete SHAP-based feature-contribution results are provided in Supplementary Table S11, and detailed database annotations and supporting information, including annotation levels, MS2 scores, *m*/*z* values, retention times, and ionization modes, are provided in Supplementary Table S12.

The relative contribution analysis further showed that metabolites accounted for most of the model contribution in all five organs, whereas microbial genera contributed a smaller proportion. This pattern was consistent with the PMI regression performance comparison, in which the integrated models generally approached the performance of the metabolomics-based models rather than showing a distinct improvement over them. Therefore, the integrated PMI regression models were mainly driven by metabolomic information, with microbial genera making a limited supplementary contribution (Figure 6F).

## Discussion

The present results show that postmortem metabolomic and microbial profiles provided different types of forensic information under the controlled heatstroke death model. Within the present RF-based selected-feature comparison framework, metabolomic features provided the main discriminative signal for distinguishing HS from C and also contributed strongly to PMI estimation within the HS group. In contrast, genus-level microbial profiles mainly reflected organ-dependent and PMI-associated postmortem succession. The integrated models achieved high performance in several organs, but the additional numerical gain from adding microbial features was limited when metabolomics-based models already performed strongly. Therefore, the term “metabolite-dominant” in this study refers to the present modeling framework and feature-selection strategy, rather than an unconditional superiority of metabolomics over microbial profiling. This distinction is important for interpreting multi-omics models in forensic settings, where a small performance gain may not justify additional sequencing cost, contamination-control requirements, computational complexity, and longer turnaround time.

The strong performance of metabolomics-based classification models may be explained by the integrative nature of tissue metabolites. Unlike a single pathological sign, a postmortem metabolic profile captures residual effects of antemortem heat exposure, terminal physiological disturbance, tissue-specific injury, and early postmortem biochemical change. This may explain why all five organs retained usable information for distinguishing HS from C. However, the observed group differences cannot be attributed to heatstroke pathophysiology alone. The HS/C comparison was confounded by terminal thermal history and agonal duration: the HS animals underwent sustained hyperthermia until death, whereas the controls were euthanized rapidly by cervical dislocation at room temperature. Although all carcasses were maintained under the same postmortem conditions, the two groups entered the postmortem period with different initial thermal states and different preceding physiological stress. These differences may have influenced early autolysis, residual enzymatic activity, metabolite turnover and degradation, and microbial proliferation or translocation. The classification models may therefore have captured a composite signal reflecting terminal hyperthermia, agonal stress, temperature-dependent early postmortem change, and heatstroke-related injury, rather than heatstroke pathophysiology alone. Accordingly, the selected features should not be interpreted as heatstroke-specific biomarkers. The difficulty of postmortem heatstroke diagnosis has been emphasized in previous clinical and forensic studies, and recent proteomic work has also suggested that molecular markers may supplement conventional morphology in heatstroke death investigation (Epstein and Yanovich, 2019; Bouchama et al., 2022; Pan et al., 2025). Transcriptomic and metabonomic studies further indicate that heat exposure can leave measurable molecular changes in liver and nervous tissues, but these findings still require cautious interpretation when transferred to forensic scenarios (Duan et al., 2026; He et al., 2023). Heatstroke-induced intestinal injury has also been described, although the present study did not directly assess whether intestinal barrier damage contributed to the observed postmortem microbial profiles (Sun et al., 2024).

These same thermal effects also create a specific methodological coupling between cause of death and postmortem progression. Because HS animals entered the postmortem period after sustained hyperthermia, HS and C samples obtained at the same chronological PMI may not represent equivalent biological stages of decomposition. Thus, HS/C classification may partly exploit divergent postmortem trajectories rather than heatstroke-specific molecular signatures.

This coupling also limits the scope of the PMI regression results. Because the regression models were trained and externally validated only in HS animals, they were calibrated to the temporal trajectory of the present thermally perturbed death model. The low MAE values of approximately 4–6 h observed for the metabolomics-based and integrated models therefore describe performance within this HS model and should not be generalized to normothermic deaths or other causes of death without independent recalibration and validation. Nevertheless, this coupling does not invalidate evaluating the two tasks within the same study because they address different questions. Classification assesses reproducible differences between the predefined HS and C groups, whereas regression assesses time-related variation within the HS condition alone. Their joint evaluation permits comparison of the relative contributions of metabolomic, microbial, and integrated features across tasks, but does not establish cause-independent diagnostic or PMI models.

The metabolomic results also showed clear PMI-related variation. The numbers and directions of increased and decreased metabolic features changed across organs and time points, indicating that the difference between HS and C was not fixed after death. This is consistent with the broader postmortem metabolomics literature, in which endogenous metabolites have been shown to change with time since death and to provide candidate information for PMI estimation (Pesko et al., 2020; Zhang et al., 2022; Lu et al., 2023; Secco et al., 2025; Steuer et al., 2024; Wu et al., 2018; Dai et al., 2019). In the present study, liver, kidney, and skeletal muscle covered the full 0–168 h range, whereas brain and cerebellum had shorter PMI coverage because of tissue decomposition and sampling limitations. The higher *R*^2^ values observed in the peripheral organs should therefore not be interpreted as evidence of their intrinsic superiority, because broader target ranges can facilitate model training and external testing, whereas restricted coverage increases uncertainty. At the same time, organs may exhibit different postmortem biochemical and microbial trajectories, all of which can affect PMI-related molecular signals (Lu et al., 2023; Lutz et al., 2020; Burcham et al., 2024). The present results therefore support organ- and PMI-window-specific model evaluation rather than a direct ranking of tissue performance across unequal temporal scales.

The microbial findings followed a different pattern from the metabolomic results. Genus-level profiles, alpha diversity, and PERMANOVA results all suggested that microbial communities changed with PMI, but stable separation between HS and C was limited. This is consistent with current postmortem microbiome research, where microbial communities are known to change during decomposition and to be influenced by body site, environment, sequencing strategy, and study design (Tozzo et al., 2022; Moitas et al., 2024; Lutz et al., 2020; Burcham et al., 2024). Studies of oral microbiota, internal organs, bone-associated communities, and decomposing animal remains have also shown that postmortem community succession can provide temporal information, although the relevant taxa and model performance vary across sampling sites and decomposition contexts (Dong et al., 2019; Javan et al., 2019; Emmons et al., 2020; Forger et al., 2019). In this study, genus-level microbial profiling was therefore more informative as a postmortem temporal signal than as an independent cause-of-death discriminator. However, this conclusion applies to the 16S rRNA-based genus-level profiles analyzed here and does not exclude the possibility that strain-level microbial translocation or functional microbial shifts could provide additional cause-of-death-related information. The relatively better performance of microbial models in some peripheral organs may reflect stronger decomposition-related community succession within the longer sampling window, rather than a heatstroke-specific microbial signature.

The integrated models provided a useful framework for comparing selected metabolomic and microbial feature layers, rather than for exhaustively testing all possible metabolite–genus combinations. In the classification task, each integrated model combined 10 selected metabolic features and 10 selected microbial genera from the same organ. Under this RF-based selected-feature framework, combining metabolites and microbial genera improved performance compared with microbial profiling alone, but provided little additional benefit over metabolomics alone. Therefore, the integrated classification results do not support routine use of 16S profiling as a necessary additional layer under the present experimental setup.

In the PMI regression task, integrated models generally approached the performance of metabolomics-based models, and SHAP analysis again showed metabolite-dominant prediction. These results suggest that integration expanded the feature space and added complementary information, but its value in the present PMI regression analysis was mainly comparative rather than practical because the principal predictive signal remained metabolic. Similar issues have been discussed in forensic multi-omics and microbial multi-omics studies, where integration can improve feature coverage or model performance, but the contribution of each omics layer may be unequal (Bonicelli et al., 2022; Li et al., 2024; Arıkan and Muth, 2023). In the absence of targeted validation or functional experiments, the integrated feature sets in the present study should be viewed as predictive combinations rather than evidence that metabolites and microbial genera jointly drive the observed forensic phenotypes.

The modeling strategy also deserves consideration. High-dimensional omics data are vulnerable to optimistic performance estimates when feature selection, model development, and validation are not properly separated. In the present classification analysis, RF-based feature selection for the single-omics models was performed within the training folds during cross-validation, and the external validation cohort was kept independent from feature selection and model training. Nevertheless, the larger metabolomic candidate feature pool may increase the chance of identifying highly discriminative features, and the metabolite-dominant pattern should therefore be interpreted within the present RF-based selected-feature design. This design is particularly relevant because recent guidance for prediction model reporting emphasizes transparent model development, appropriate validation, and clear reporting of model use cases (Collins et al., 2024). Previous methodological reviews have also shown that more complex machine learning methods do not automatically outperform simpler approaches if study design, validation, and sample size are not adequate (Christodoulou et al., 2019). In forensic medicine, the use of artificial intelligence and machine learning remains largely in the research stage, and a recent scoping review and subsequent critical commentary have emphasized that model interpretability, validation, and practical implementation require careful assessment before routine application (Tournois et al., 2024; Ondruschka et al., 2024). Accordingly, RF importance and SHAP values in this study should be interpreted as model-level explanations, not as evidence of biological causality. This is particularly important for imputed metabolomic features, because imputation may influence the apparent split points in tree-based models and the estimated contribution of individual variables.

Several limitations should be considered when interpreting these results. First, as discussed above, terminal thermal history and agonal duration were confounded with HS/C group assignment. The present design therefore cannot isolate heatstroke-related pathology from terminal hyperthermia, agonal stress, or temperature-dependent early postmortem change, and the HS-only PMI models cannot be generalized to normothermic deaths. Future studies should include a normothermic non-instantaneous death control with a comparable agonal duration, a heat-exposed survivor or sham group, and, where feasible, a control matched for terminal body temperature. Second, the study was based on a controlled rat model, which cannot fully reproduce the heterogeneity of human heatstroke deaths. Real forensic cases may involve different environmental exposures, clothing, body habitus, underlying disease, resuscitation, medication, and uneven cooling or decomposition. More specifically, species-related differences in body mass, surface-area-to-volume ratio, and cooling kinetics may alter the rate of postmortem biochemical change, such that the same chronological PMI may not represent an equivalent biological stage in rats and humans. In addition, the present experiment used precisely known and predefined sampling time points, whereas PMIs in forensic casework are often uncertain, interval-based, or censored and may be unevenly distributed across the forensically relevant time range. Consequently, models developed in rats cannot be assumed to retain their calibration, prediction error, selected-feature relationships, or relative organ performance when applied directly to human tissues. Consistent with this concern, a recent cross-species postmortem metabolomics study comparing ovine and human aqueous humor found qualitative overlap in many identified metabolites, but also species-dependent quantitative differences and lower PMI-prediction accuracy in the human model, indicating that translation from animal data may be partial rather than direct (Chighine et al., 2025). Determining whether the relative organ performance and metabolite-dominant pattern observed in the present study persist in human casework will require analyses of comparable human organs across forensically relevant PMI ranges using harmonized analytical workflows, explicit consideration of PMI uncertainty, model retraining or recalibration using human data, and independent validation in a separate human cohort. Third, the postmortem environment was controlled, whereas actual death scenes may include fluctuating temperature and humidity, insect activity, soil contact, water exposure, delayed discovery, and other uncontrolled factors. Fourth, the brain and cerebellum had shorter PMI coverage than liver, kidney, and skeletal muscle, which may have affected the stability of central nervous tissue models. Fifth, the independent external-validation subsets were relatively small, particularly for the brain and cerebellum and for the PMI-stratified classification analyses. At each PMI point, the stratified external-validation analysis included only three HS and three C samples. Consequently, the high or apparently perfect performance observed in some organ- and PMI-specific subsets should be interpreted cautiously, as these small sample sizes result in considerable uncertainty in performance estimates and provide only limited evidence of model generalizability. Larger independent validation cohorts are required to obtain more precise performance estimates and to further assess model robustness. Sixth, the conclusion that postmortem microbial profiles had limited discriminative value for distinguishing HS from C should be interpreted in light of the use of 16S rRNA sequencing. This approach provides genus-level microbial information but has limited resolution for strain-level variation and functional inference. Therefore, strain-level pathogenic translocation or functional microbial shifts might have been missed, and marker-gene sequencing cannot fully replace shotgun metagenomics or direct functional profiling (Knight et al., 2018). Finally, untargeted metabolomics is affected by annotation confidence, matrix effects, missing values, and platform-specific variation. Although annotation confidence was categorized using an MSI-referenced scheme, no additional targeted confirmation with authentic standards was performed for the features reported in this study. Therefore, Level 2 and Level 3 names should be interpreted as putative annotations rather than definitive evidence of compound identity or biological origin. To avoid overinterpretation, the features displayed in Figure 6E were labeled using organ-specific feature IDs, with the corresponding candidate annotations and confidence information provided in Supplementary Table S12. In this study, remaining missing metabolomic values after quality filtering were imputed using half of the minimum positive value for the corresponding feature. Although this approach reduces data sparsity and allows statistical modeling, postmortem metabolic changes may include true biological appearance or disappearance of specific metabolites. Therefore, this imputation strategy may dampen temporal variance in some features and should be considered when interpreting feature importance and SHAP-based explanations. Future studies should include larger sample sizes, human cadaver validation, broader environmental conditions, and standardized analytical pipelines.

In summary, postmortem metabolomic and microbial profiles provided complementary but unequal information in this controlled comparison. The classification models distinguished the present rat model of heatstroke death from controls euthanized by cervical dislocation. However, because terminal thermal history and agonal duration were confounded with HS/C group assignment, this performance should not be interpreted as evidence of heatstroke-specific discrimination. Similarly, the PMI regression results describe temporal prediction within the present HS model and should not be extrapolated to normothermic deaths. Within this experimental framework, metabolomic features provided the main predictive information, whereas microbial profiling mainly reflected PMI-associated community succession. Adding microbial profiles to metabolomics produced limited additional practical value for the classification task, and integrated modeling should therefore be viewed mainly as a comparative framework rather than a necessary routine approach. The results support the use of multi-organ and multi-feature comparisons in forensic omics research, while also emphasizing that organ selection, PMI range, temporal coverage, external validation, and cautious interpretation remain essential before translation to forensic practice.

## Data Availability

The raw 16S rRNA amplicon sequencing data have been deposited in the NCBI Sequence Read Archive (SRA) under BioProject accession PRJNA1519882 (https://www.ncbi.nlm.nih.gov/bioproject/PRJNA1519882). The processed metabolomic abundance matrices and genus-level microbial abundance tables used for model development and independent external validation in the HS/C classification and HS-only PMI regression analyses are publicly available in Zenodo at https://doi.org/10.5281/zenodo.22139280.
