# Supplementary material for: Comparative evaluation of metabolomic, microbial, and integrated features for distinguishing a rat model of heatstroke death from cervical-dislocation controls and estimating postmortem interval across organs
Source: Front Microbiol. 2026 Sep 29;17:1897987. doi: 10.3389/fmicb.2026.1897987 (PMC13658517; doi:10.3389/fmicb.2026.1897987)
Supplement: Supplementary file 1 [file Supplementary_File_1.DOCX]

Supplementary Material

**Supplementary Figure S1. OPLS-DA score plots of metabolomic profiles across five organs.** (A–E) OPLS-DA score plots showing the separation between the heatstroke death group (HS) and the control group (C) based on untargeted metabolomic profiles in the brain (A), cerebellum (B), liver (C), kidney (D), and skeletal muscle (E). HS, heatstroke death group; C, control group; OPLS-DA, orthogonal partial least squares discriminant analysis.

**Supplementary Figure S2. Permutation validation of OPLS-DA models across five organs.** (A–E) Corresponding 200-permutation validation plots for the OPLS-DA models in the brain (A), cerebellum (B), liver (C), kidney (D), and skeletal muscle (E). Red circles indicate R², and blue circles indicate Q^2^. The low or negative Q^2^ intercepts indicate that the OPLS-DA models were not obviously overfitted. HS, heatstroke death group; C, control group; OPLS-DA, orthogonal partial least squares discriminant analysis.

**Supplementary Figure S3. Beta-diversity patterns of postmortem microbial communities across five organs.** (A–E) Principal coordinate analysis (PCoA) plots based on Bray–Curtis distances of genus-level microbial profiles in the brain (A), cerebellum (B), liver (C), kidney (D), and skeletal muscle (E). Samples are shown according to group and postmortem interval. These plots were used to visualize organ-specific and PMI-associated shifts in postmortem microbial community structure. HS, heatstroke death group; C, control group; PMI, postmortem interval; PCoA, principal coordinate analysis.

**Supplementary Table S1. Composition of the task-specific independent external-validation subsets used for HS/C classification and PMI regression.**

| **Task** | **Organ** | **Groups included** | **PMI design** | **PMI range** | **External validation sample size** |
| --- | --- | --- | --- | --- | --- |
| HS/C classification | Brain | HS and C | Fixed matched PMI points | 0, 12, 24, 48, 72 h | 15 HS, 15 C |
| HS/C classification | Cerebellum | HS and C | Matched-PMI subset | 0, 12, 24, 48 h | 12 HS, 12 C |
| HS/C classification | Liver | HS and C | Fixed matched PMI points | 0, 12, 24, 48, 72, 96, 120, 144, 168 h | 27 HS, 27 C |
| HS/C classification | Kidney | HS and C | Fixed matched PMI points | 0, 12, 24, 48, 72, 96, 120, 144, 168 h | 27 HS, 27 C |
| HS/C classification | Skeletal muscle | HS and C | Fixed matched PMI points | 0, 12, 24, 48, 72, 96, 120, 144, 168 h | 27 HS, 27 C |
| PMI regression | Brain | HS only | Random, non-fixed PMIs | 0–72 h | 15 HS |
| PMI regression | Cerebellum | HS only | Random, non-fixed PMIs | 0–48 h | 12 HS |
| PMI regression | Liver | HS only | Random, non-fixed PMIs | 0–168 h | 27 HS |
| PMI regression | Kidney | HS only | Random, non-fixed PMIs | 0–168 h | 27 HS |
| PMI regression | Skeletal muscle | HS only | Random, non-fixed PMIs | 0–168 h | 27 HS |

Note: For HS/C classification, external validation was performed using fixed PMI-matched HS and C samples. For PMI regression, external validation included independent HS samples collected at random, non-fixed PMIs within the organ-specific available ranges. Integrated models included only samples with paired metabolomic and microbial profiles from the same Sample ID. HS, heatstroke death group; C, control group; PMI, postmortem interval.

| **Organ** | **Feature strategy** | **Candidate feature pool** | **Selected input features** | **Classifier** |
| --- | --- | --- | --- | --- |
| Brain | Metabolomics | 2372 metabolic features | 10 metabolic features | RF |
| Brain | Microbial profiling | 177 genera | 10 genera | RF |
| Brain | Integrated metabolite–genus features | 2372 metabolic features + 177 genera | 20 features, including 10 metabolites and 10 genera | RF |
| Cerebellum | Metabolomics | 2247 metabolic features | 10 metabolic features | RF |
| Cerebellum | Microbial profiling | 163 genera | 10 genera | RF |
| Cerebellum | Integrated metabolite–genus features | 2247 metabolic features + 163 genera | 20 features, including 10 metabolites and 10 genera | RF |
| Liver | Metabolomics | 3032 metabolic features | 10 metabolic features | RF |
| Liver | Microbial profiling | 175 genera | 10 genera | RF |
| Liver | Integrated metabolite–genus features | 3032 metabolic features + 175 genera | 20 features, including 10 metabolites and 10 genera | RF |
| Kidney | Metabolomics | 2992 metabolic features | 10 metabolic features | RF |
| Kidney | Microbial profiling | 230 genera | 10 genera | RF |
| Kidney | Integrated metabolite–genus features | 2992 metabolic features + 230 genera | 20 features, including 10 metabolites and 10 genera | RF |
| Skeletal muscle | Metabolomics | 3051 metabolic features | 10 metabolic features | RF |
| Skeletal muscle | Microbial profiling | 165 genera | 10 genera | RF |
| Skeletal muscle | Integrated metabolite–genus features | 3051 metabolic features + 165 genera | 20 features, including 10 metabolites and 10 genera | RF |

**Supplementary Table S2. Candidate feature-pool sizes and selected input-feature numbers used for HS/C classification models.**

**Note:** Candidate feature pool indicates the number of available features before RF-based feature selection in each organ. Metabolic feature-pool sizes refer to MS2-annotated metabolic features retained after quality control and annotation. Microbial feature-pool sizes refer to genus-level microbial features used for machine learning. In the single-omics classification models, the 10 highest-ranked metabolic features or genus-level microbial features were selected for each organ. In the integrated metabolite–genus models, the selected 10 metabolic features and 10 microbial genera from the same organ were combined, resulting in 20 input features. RF was used as the common classifier for all three feature strategies. RF, random forest; HS, heatstroke death group; C, control group.

**Supplementary Table S3. Statistical summary of alpha diversity comparisons across organs, groups, and postmortem intervals.**

| **Organ** | **Alpha diversity index** | **Time effect within HS group, P value** | **Time effect within C group, P value** | **Significant HS vs C PMI points** |
| --- | --- | --- | --- | --- |
| Brain | Chao1 | 0.241 | 0.270 | None |
|  | Shannon | 0.103 | 0.313 | None |
|  | Simpson | 0.031 | 0.056 | None |
|  | Pielou_e | 0.044 | 0.074 | None |
| Cerebellum | Chao1 | 0.738 | 0.592 | None |
|  | Shannon | 0.161 | 0.118 | None |
|  | Simpson | 0.139 | 0.149 | None |
|  | Pielou_e | 0.046 | 0.098 | None |
| Liver | Chao1 | 0.003 | 0.001 | None |
|  | Shannon | 0.022 | 0.006 | 24 h, 168 h |
|  | Simpson | 0.013 | 0.007 | None |
|  | Pielou_e | <0.001 | 0.007 | None |
| Kidney | Chao1 | 0.039 | 0.002 | None |
|  | Shannon | 0.001 | 0.110 | None |
|  | Simpson | 0.001 | 0.027 | None |
|  | Pielou_e | 0.001 | 0.006 | None |
| Skeletal muscle | Chao1 | 0.012 | 0.019 | None |
|  | Shannon | 0.001 | 0.002 | None |
|  | Simpson | <0.001 | 0.001 | None |
|  | Pielou_e | 0.001 | 0.001 | None |

**Note:** Alpha diversity indices included Chao1, Shannon, Simpson, and Pielou_e. Time effects within the HS and C groups indicate comparisons across available postmortem intervals within each organ. Significant HS vs C PMI points indicate matched postmortem intervals at which a significant difference between the two groups was observed after multiple-comparison correction. P values reported as 0.000 in the original statistical output are shown as <0.001. HS, heatstroke death group; C, control group; PMI, postmortem interval.

**Supplementary Table S4. PERMANOVA results of microbial beta diversity across organs and postmortem intervals based on Bray–Curtis distances.**

| **Organ** | **Comparison range** | **Sample size** | **Number of groups** | **Pseudo-F** | **P value** | **Permutations** |
| --- | --- | --- | --- | --- | --- | --- |
| Brain | HS group | 24 | 5 | 3.214 | 0.001 | 999 |
|  | C group | 25 | 5 | 2.051 | 0.002 | 999 |
|  | Stratified HS+C group | 49 | 10 | 2.607 | 0.001 | 999 |
| Cerebellum | HS group | 20 | 4 | 1.844 | 0.012 | 999 |
|  | C group | 24 | 5 | 3.399 | 0.001 | 999 |
|  | Stratified HS+C group | 44 | 9 | 2.537 | 0.001 | 999 |
| Liver | HS group | 45 | 9 | 3.023 | 0.001 | 999 |
|  | C group | 45 | 9 | 2.839 | 0.001 | 999 |
|  | Stratified HS+C group | 90 | 18 | 3.686 | 0.001 | 999 |
| Kidney | HS group | 45 | 9 | 2.961 | 0.001 | 999 |
|  | C group | 45 | 9 | 2.839 | 0.001 | 999 |
|  | Stratified HS+C group | 90 | 18 | 4.229 | 0.001 | 999 |
| Skeletal muscle | HS group | 45 | 9 | 6.424 | 0.001 | 999 |
|  | C group | 45 | 9 | 4.671 | 0.001 | 999 |
|  | Stratified HS+C group | 90 | 18 | 5.539 | 0.001 | 999 |

**Note:**
Beta diversity was calculated using Bray–Curtis distances based on genus-level microbial profiles, and PERMANOVA was performed to test differences in microbial community structure across postmortem intervals. The HS group and C group indicate within-group comparisons across available PMI points for each organ. The stratified HS+C group indicates the combined analysis of HS and C samples within the same organ, with group and PMI strata considered together. In brain samples, the PMI range was 0–72 h for both HS and C groups. In cerebellum samples, the PMI range was 0–48 h for the HS group and 0–72 h for the C group. In liver, kidney, and skeletal muscle samples, the PMI range was 0–168 h for both groups. HS, heatstroke death group; C, control group; PMI, postmortem interval; PERMANOVA, permutational multivariate analysis of variance.

**Supplementary Table S5. Fold-level cross-validation performance of HS/C classification models across five organs and feature strategies.**

| **Organ** | **Feature strategy** | **Accuracy (mean ± SD)** | **Precision (mean ± SD)** | **Recall (mean ± SD)** | **F1-score (mean ± SD)** | **Fold-level AUC (mean ± SD)** |
| --- | --- | --- | --- | --- | --- | --- |
| Brain | Metabolomics | 0.943 ± 0.078 | 0.961 ± 0.081 | 0.928 ± 0.114 | 0.940 ± 0.083 | 0.994 ± 0.025 |
| Brain | Microbial profiling | 0.719 ± 0.115 | 0.707 ± 0.138 | 0.802 ± 0.124 | 0.743 ± 0.100 | 0.847 ± 0.105 |
| Brain | Integrated metabolite–genus features | 0.951 ± 0.066 | 0.949 ± 0.083 | 0.960 ± 0.082 | 0.952 ± 0.066 | 0.994 ± 0.025 |
| Cerebellum | Metabolomics | 0.985 ± 0.041 | 0.992 ± 0.040 | 0.980 ± 0.069 | 0.984 ± 0.044 | 0.998 ± 0.013 |
| Cerebellum | Microbial profiling | 0.745 ± 0.127 | 0.730 ± 0.137 | 0.820 ± 0.198 | 0.757 ± 0.129 | 0.861 ± 0.124 |
| Cerebellum | Integrated metabolite–genus features | 0.990 ± 0.035 | 0.984 ± 0.055 | 1.000 ± 0.000 | 0.991 ± 0.031 | 0.995 ± 0.017 |
| Liver | Metabolomics | 0.882 ± 0.070 | 0.901 ± 0.096 | 0.876 ± 0.121 | 0.880 ± 0.076 | 0.966 ± 0.033 |
| Liver | Microbial profiling | 0.836 ± 0.059 | 0.830 ± 0.078 | 0.853 ± 0.062 | 0.839 ± 0.055 | 0.900 ± 0.036 |
| Liver | Integrated metabolite–genus features | 0.949 ± 0.051 | 0.958 ± 0.069 | 0.947 ± 0.079 | 0.949 ± 0.050 | 0.984 ± 0.026 |
| Kidney | Metabolomics | 0.980 ± 0.027 | 0.988 ± 0.033 | 0.973 ± 0.048 | 0.980 ± 0.028 | 1.000 ± 0.000 |
| Kidney | Microbial profiling | 0.762 ± 0.076 | 0.762 ± 0.092 | 0.787 ± 0.157 | 0.762 ± 0.090 | 0.868 ± 0.065 |
| Kidney | Integrated metabolite–genus features | 0.969 ± 0.040 | 0.992 ± 0.028 | 0.947 ± 0.079 | 0.967 ± 0.043 | 1.000 ± 0.002 |
| Skeletal muscle | Metabolomics | 0.904 ± 0.049 | 0.902 ± 0.068 | 0.916 ± 0.080 | 0.905 ± 0.050 | 0.969 ± 0.028 |
| Skeletal muscle | Microbial profiling | 0.820 ± 0.081 | 0.829 ± 0.101 | 0.822 ± 0.101 | 0.821 ± 0.078 | 0.907 ± 0.054 |
| Skeletal muscle | Integrated metabolite–genus features | 0.942 ± 0.047 | 0.946 ± 0.061 | 0.942 ± 0.065 | 0.942 ± 0.047 | 0.987 ± 0.016 |

Note: Classification models were constructed to distinguish the heatstroke death group from the control group in each organ. Accuracy, precision, recall, F1-score, and fold-level AUC are shown as mean ± standard deviation across the 25 held-out validation folds generated by repeated five-fold cross-validation with five repeats in the model-development cohort. Fold assignment was balanced jointly by HS/C group and PMI stratum, and scaling, RF-based feature ranking, and feature selection were performed within each training fold. The fold-level AUC values summarize AUCs calculated within individual validation folds and are therefore distinct from the aggregated sample-level OOF AUC values reported in the main text and shown in Fig. 4A–C. HS was treated as the positive class. Integrated metabolite–genus features indicate models constructed by combining 10 selected metabolic features and 10 selected genus-level microbial features. HS, heatstroke death group; C, control group; PMI, postmortem interval; RF, random forest; SD, standard deviation; OOF, out-of-fold; AUC, area under the receiver operating characteristic curve.

**Supplementary Table S6. Independent external validation confusion-matrix metrics of HS/C classification models across organs and feature strategies.**

| **Organ** | **Feature strategy** | **n (HS/C)** | **TP** | **FN** | **FP** | **TN** | **Acc.** | **Prec.** | **Recall** | **Spec.** | **F1** |
| --- | --- | --- | --- | --- | --- | --- | --- | --- | --- | --- | --- |
| Brain | Metabolomics | 30 (15/15) | 15 | 0 | 0 | 15 | 1.000 | 1.000 | 1.000 | 1.000 | 1.000 |
| Brain | Microbial profiling | 30 (15/15) | 15 | 0 | 3 | 12 | 0.900 | 0.833 | 1.000 | 0.800 | 0.909 |
| Brain | Integrated metabolite–genus features | 30 (15/15) | 15 | 0 | 0 | 15 | 1.000 | 1.000 | 1.000 | 1.000 | 1.000 |
| Cerebellum | Metabolomics | 24 (12/12) | 12 | 0 | 0 | 12 | 1.000 | 1.000 | 1.000 | 1.000 | 1.000 |
| Cerebellum | Microbial profiling | 24 (12/12) | 12 | 0 | 0 | 12 | 1.000 | 1.000 | 1.000 | 1.000 | 1.000 |
| Cerebellum | Integrated metabolite–genus features | 24 (12/12) | 12 | 0 | 0 | 12 | 1.000 | 1.000 | 1.000 | 1.000 | 1.000 |
| Liver | Metabolomics | 54 (27/27) | 27 | 0 | 0 | 27 | 1.000 | 1.000 | 1.000 | 1.000 | 1.000 |
| Liver | Microbial profiling | 54 (27/27) | 23 | 4 | 0 | 27 | 0.926 | 1.000 | 0.852 | 1.000 | 0.920 |
| Liver | Integrated metabolite–genus features | 54 (27/27) | 27 | 0 | 0 | 27 | 1.000 | 1.000 | 1.000 | 1.000 | 1.000 |
| Kidney | Metabolomics | 54 (27/27) | 27 | 0 | 0 | 27 | 1.000 | 1.000 | 1.000 | 1.000 | 1.000 |
| Kidney | Microbial profiling | 54 (27/27) | 22 | 5 | 2 | 25 | 0.870 | 0.917 | 0.815 | 0.926 | 0.863 |
| Kidney | Integrated metabolite–genus features | 54 (27/27) | 27 | 0 | 0 | 27 | 1.000 | 1.000 | 1.000 | 1.000 | 1.000 |
| Skeletal muscle | Metabolomics | 54 (27/27) | 27 | 0 | 0 | 27 | 1.000 | 1.000 | 1.000 | 1.000 | 1.000 |
| Skeletal muscle | Microbial profiling | 54 (27/27) | 25 | 2 | 0 | 27 | 0.963 | 1.000 | 0.926 | 1.000 | 0.962 |
| Skeletal muscle | Integrated metabolite–genus features | 54 (27/27) | 27 | 0 | 0 | 27 | 1.000 | 1.000 | 1.000 | 1.000 | 1.000 |

Note: External validation was performed using independent organ-specific PMI-matched HS/C classification subsets, with HS treated as the positive class. Final class labels were assigned using a predicted-probability threshold of 0.5. For the cerebellum, the external validation confusion matrices were calculated using the matched-PMI subset restricted to 0, 12, 24, and 48 h, with 12 HS and 12 C samples; unmatched cerebellar C samples at 72 h and samples outside the matched-PMI structure were excluded. Integrated metabolite–genus models were evaluated using samples with paired metabolomic and microbial profiles from the same Sample ID. TP, true positive; FN, false negative; FP, false positive; TN, true negative; Acc., accuracy; Prec., precision; Spec., specificity; F1, F1-score. Accuracy was calculated as (TP+TN)/(TP+FN+FP+TN), precision as TP/(TP+FP), recall/sensitivity as TP/(TP+FN), specificity as TN/(TN+FP), and F1-score as 2TP/(2TP+FP+FN). HS, heatstroke death group; C, control group; PMI, postmortem interval.

**Supplementary Table S7. PMI-stratified aggregated out-of-fold performance of HS/C classification models across organs and feature strategies.**

**Note:** Aggregated OOF probabilities were calculated at the sample level by averaging the five repeat-specific OOF probabilities obtained for each sample during repeated five-fold cross-validation. Metrics were then calculated separately within each available PMI stratum using a predicted-probability threshold of 0.5, with HS treated as the positive class. Most development-cohort PMI strata contained 5 HS and 5 C samples; the brain at 72 h contained 4 HS and 4 C samples. PMI points not available for a given organ were omitted. Integrated indicates models using 10 selected metabolic features and 10 selected genus-level microbial features. HS, heatstroke death group; C, control group; PMI, postmortem interval; OOF, out-of-fold; TP, true positive; FN, false negative; FP, false positive; TN, true negative; AUC, area under the receiver operating characteristic curve.

**Supplementary Table S8. PMI-stratified external-validation performance of HS/C classification models across organs and feature strategies.**

**Note:** External validation was performed using independent organ-specific HS/C samples matched by PMI. Metrics were calculated separately within each available PMI stratum using a predicted-probability threshold of 0.5, with HS treated as the positive class. Each reported PMI stratum contained 3 HS and 3 C samples. Cerebellum analyses were restricted to the matched PMI points of 0, 12, 24, and 48 h. Because each PMI-specific external-validation subset contained only six samples, these results should be interpreted descriptively. Integrated indicates models using 10 selected metabolic features and 10 selected genus-level microbial features. HS, heatstroke death group; C, control group; PMI, postmortem interval; TP, true positive; FN, false negative; FP, false positive; TN, true negative; AUC, area under the receiver operating characteristic curve.

**Supplementary Table S9. Top selected features, RF importance, and directionality in integrated classification models across organs.**

**Note:** Selected features were derived from the final integrated classification models constructed using PMI-matched HS/C model-development datasets. Each organ-specific integrated model included 10 selected metabolic features and 10 selected genus-level microbial features. Features are ranked within each organ according to random forest importance. The log2(HS/C) value was calculated from the unscaled abundance values in the PMI-matched model-development cohort as log2[(mean abundance in HS + 1×10^-6^)/(mean abundance in C + 1×10^-6^)]. Positive values indicate higher relative abundance or level in the HS group, whereas negative values indicate higher relative abundance or level in the C group.

**Supplementary Table S10. Cross-validation and external validation performance of PMI regression models across organs and feature strategies.**

| **Organ** | **Feature strategy** | **CV MAE (h)** | **CV RMSE (h)** | **CV MAPE (%)** | **CV R^2^** | **External MAE (h)** | **External RMSE (h)** | **External MAPE (%)** | **External R^2^** |
| --- | --- | --- | --- | --- | --- | --- | --- | --- | --- |
| Brain | Metabolomics | 3.52 | 5.78 | 16.33 | 0.947 | 6.09 | 12.80 | 20.00 | 0.763 |
|  | Microbial profiling | 12.23 | 15.49 | 57.67 | 0.615 | 20.69 | 33.43 | 43.17 | -0.619 |
|  | Integrated metabolite–genus features | 3.56 | 5.80 | 16.39 | 0.946 | 6.44 | 13.17 | 20.60 | 0.749 |
| Cerebellum | Metabolomics | 2.98 | 5.44 | 18.23 | 0.906 | 4.00 | 6.35 | 26.74 | 0.787 |
|  | Microbial profiling | 7.17 | 8.60 | 35.50 | 0.765 | 4.00 | 6.35 | 26.74 | 0.787 |
|  | Integrated metabolite–genus features | 3.00 | 5.32 | 18.41 | 0.910 | 4.13 | 6.36 | 27.36 | 0.786 |
| Liver | Metabolomics | 11.58 | 14.50 | 22.75 | 0.934 | 4.48 | 8.22 | 11.08 | 0.977 |
|  | Microbial profiling | 28.16 | 38.22 | 47.86 | 0.544 | 4.79 | 8.59 | 9.63 | 0.975 |
|  | Integrated metabolite–genus features | 11.33 | 14.43 | 21.80 | 0.935 | 4.94 | 8.25 | 11.87 | 0.977 |
| Kidney | Metabolomics | 10.24 | 14.45 | 17.43 | 0.935 | 4.69 | 8.55 | 10.18 | 0.975 |
|  | Microbial profiling | 23.72 | 33.21 | 38.85 | 0.655 | 6.47 | 15.47 | 12.81 | 0.919 |
|  | Integrated metabolite–genus features | 9.05 | 12.80 | 14.42 | 0.949 | 4.54 | 8.22 | 10.28 | 0.977 |
| Skeletal muscle | Metabolomics | 8.93 | 12.77 | 16.54 | 0.949 | 4.24 | 7.84 | 9.53 | 0.979 |
|  | Microbial profiling | 32.10 | 44.54 | 50.39 | 0.380 | 4.04 | 7.99 | 9.32 | 0.978 |
|  | Integrated metabolite–genus features | 8.76 | 12.32 | 15.66 | 0.953 | 4.06 | 7.81 | 9.39 | 0.979 |

**Note:** PMI regression models were constructed only within the heatstroke death group. Model performance was evaluated by cross-validation in the model development cohort and by independent external validation. MAPE was calculated after excluding 0 h samples. Integrated metabolite–genus features indicate models constructed by combining selected metabolomic features and genus-level microbial features. CV, cross-validation; PMI, postmortem interval; MAE, mean absolute error; RMSE, root mean square error; MAPE, mean absolute percentage error; R², coefficient of determination.

**Supplementary Table S11. SHAP-based feature contribution in integrated PMI regression models across organs.**

**Note:** The complete table is provided as a separate Excel file. SHAP analysis was performed on the integrated PMI regression models to estimate the contribution of each selected feature to PMI prediction. Features are ranked within each organ by mean absolute SHAP value. Feature relative contribution was calculated as the mean absolute SHAP value of each feature divided by the sum of mean absolute SHAP values within the same organ. Feature type contribution was calculated by summing the mean absolute SHAP values of metabolites or microbial genera within each organ. PMI, postmortem interval; SHAP, Shapley additive explanations.

**Supplementary Table S12. Annotation information for the top three SHAP-ranked features from the integrated PMI regression model in each organ.**

**Note:** Feature IDs correspond to the labels used in Figure 6E. Annotation confidence followed the scheme reported in the metabolomics output. Level 1 indicates matching of MS1, MS2, and retention time against the vendor’s in-house authentic-standard library; Level 2 indicates MS1 and MS2 database matching; and Level 3 indicates theoretical-database annotation based on MS1, MS2, and predicted retention time. Level 3.1 and Level 3.2 are retained as reported in the vendor output, for which separate subclass definitions were not provided. No separate targeted confirmation with authentic standards was performed in the present study; therefore, Level 2 and Level 3 names should be interpreted as putative annotations. Higher MS2 scores indicate better spectral matching. POS and NEG denote positive- and negative-ion modes, respectively. Feature IDs are organ-specific and should not be compared across organs. PMI, postmortem interval; SHAP, SHapley Additive exPlanations.
