# Supplementary material for: Comparative evaluation of metabolomic, microbial, and integrated features for distinguishing a rat model of heatstroke death from cervical-dislocation controls and estimating postmortem interval across organs
Source: Front Microbiol. 2026 Sep 29;17:1897987. doi: 10.3389/fmicb.2026.1897987 (PMC13658517; doi:10.3389/fmicb.2026.1897987)
Supplement: Supplementary file 2 [file Table_7.DOCX]

**Supplementary Table S7. PMI-stratified aggregated OOF performance of HS/C classification models across organs and feature strategies.**

| **Organ** | **Feature strategy** | **PMI (h)** | **n (HS/C)** | **TP** | **FN** | **FP** | **TN** | **Accuracy** | **Precision** | **Sensitivity** | **Specificity** | **F1-score** | **AUC** |
| --- | --- | --- | --- | --- | --- | --- | --- | --- | --- | --- | --- | --- | --- |
| Brain | Metabolomics | **0** | 10 (5/5) | 5 | 0 | 0 | 5 | 1.000 | 1.000 | 1.000 | 1.000 | 1.000 | 1.000 |
| Brain | Metabolomics | 12 | 10 (5/5) | 4 | 1 | 0 | 5 | 0.900 | 1.000 | 0.800 | 1.000 | 0.889 | 1.000 |
| Brain | Metabolomics | 24 | 10 (5/5) | 5 | 0 | 1 | 4 | 0.900 | 0.833 | 1.000 | 0.800 | 0.909 | 1.000 |
| Brain | Metabolomics | 48 | 10 (5/5) | 5 | 0 | 0 | 5 | 1.000 | 1.000 | 1.000 | 1.000 | 1.000 | 1.000 |
| Brain | Metabolomics | **72** | 8 (4/4) | 3 | 1 | 0 | 4 | 0.875 | 1.000 | 0.750 | 1.000 | 0.857 | 1.000 |
| Brain | Microbial profiling | **0** | 10 (5/5) | 5 | 0 | 0 | 5 | 1.000 | 1.000 | 1.000 | 1.000 | 1.000 | 1.000 |
| Brain | Microbial profiling | 12 | 10 (5/5) | 3 | 2 | 1 | 4 | 0.700 | 0.750 | 0.600 | 0.800 | 0.667 | 0.720 |
| Brain | Microbial profiling | 24 | 10 (5/5) | 4 | 1 | 3 | 2 | 0.600 | 0.571 | 0.800 | 0.400 | 0.667 | 0.640 |
| Brain | Microbial profiling | 48 | 10 (5/5) | 5 | 0 | 0 | 5 | 1.000 | 1.000 | 1.000 | 1.000 | 1.000 | 1.000 |
| Brain | Microbial profiling | **72** | 8 (4/4) | 3 | 1 | 4 | 0 | 0.375 | 0.429 | 0.750 | 0.000 | 0.545 | 0.750 |
| Brain | Integrated | **0** | 10 (5/5) | 5 | 0 | 0 | 5 | 1.000 | 1.000 | 1.000 | 1.000 | 1.000 | 1.000 |
| Brain | Integrated | 12 | 10 (5/5) | 4 | 1 | 0 | 5 | 0.900 | 1.000 | 0.800 | 1.000 | 0.889 | 1.000 |
| Brain | Integrated | 24 | 10 (5/5) | 5 | 0 | 1 | 4 | 0.900 | 0.833 | 1.000 | 0.800 | 0.909 | 1.000 |
| Brain | Integrated | 48 | 10 (5/5) | 5 | 0 | 0 | 5 | 1.000 | 1.000 | 1.000 | 1.000 | 1.000 | 1.000 |
| Brain | Integrated | **72** | 8 (4/4) | 3 | 1 | 0 | 4 | 0.875 | 1.000 | 0.750 | 1.000 | 0.857 | 1.000 |
| Cerebellum | Metabolomics | **0** | 10 (5/5) | 5 | 0 | 0 | 5 | 1.000 | 1.000 | 1.000 | 1.000 | 1.000 | 1.000 |
| Cerebellum | Metabolomics | 12 | 10 (5/5) | 5 | 0 | 0 | 5 | 1.000 | 1.000 | 1.000 | 1.000 | 1.000 | 1.000 |
| Cerebellum | Metabolomics | 24 | 10 (5/5) | 5 | 0 | 0 | 5 | 1.000 | 1.000 | 1.000 | 1.000 | 1.000 | 1.000 |
| Cerebellum | Metabolomics | 48 | 10 (5/5) | 5 | 0 | 0 | 5 | 1.000 | 1.000 | 1.000 | 1.000 | 1.000 | 1.000 |
| Cerebellum | Microbial profiling | **0** | 10 (5/5) | 4 | 1 | 3 | 2 | 0.600 | 0.571 | 0.800 | 0.400 | 0.667 | 0.880 |
| Cerebellum | Microbial profiling | 12 | 10 (5/5) | 5 | 0 | 4 | 1 | 0.600 | 0.556 | 1.000 | 0.200 | 0.714 | 0.760 |
| Cerebellum | Microbial profiling | 24 | 10 (5/5) | 4 | 1 | 0 | 5 | 0.900 | 1.000 | 0.800 | 1.000 | 0.889 | 0.920 |
| Cerebellum | Microbial profiling | 48 | 10 (5/5) | 4 | 1 | 0 | 5 | 0.900 | 1.000 | 0.800 | 1.000 | 0.889 | 1.000 |
| Cerebellum | Integrated | **0** | 10 (5/5) | 5 | 0 | 0 | 5 | 1.000 | 1.000 | 1.000 | 1.000 | 1.000 | 1.000 |
| Cerebellum | Integrated | 12 | 10 (5/5) | 5 | 0 | 0 | 5 | 1.000 | 1.000 | 1.000 | 1.000 | 1.000 | 1.000 |
| Cerebellum | Integrated | 24 | 10 (5/5) | 5 | 0 | 0 | 5 | 1.000 | 1.000 | 1.000 | 1.000 | 1.000 | 1.000 |
| Cerebellum | Integrated | 48 | 10 (5/5) | 5 | 0 | 1 | 4 | 0.900 | 0.833 | 1.000 | 0.800 | 0.909 | 1.000 |
| Liver | Metabolomics | **0** | 10 (5/5) | 4 | 1 | 0 | 5 | 0.900 | 1.000 | 0.800 | 1.000 | 0.889 | 1.000 |
| Liver | Metabolomics | 12 | 10 (5/5) | 5 | 0 | 0 | 5 | 1.000 | 1.000 | 1.000 | 1.000 | 1.000 | 1.000 |
| Liver | Metabolomics | 24 | 10 (5/5) | 5 | 0 | 0 | 5 | 1.000 | 1.000 | 1.000 | 1.000 | 1.000 | 1.000 |
| Liver | Metabolomics | 48 | 10 (5/5) | 5 | 0 | 0 | 5 | 1.000 | 1.000 | 1.000 | 1.000 | 1.000 | 1.000 |
| Liver | Metabolomics | **72** | 10 (5/5) | 3 | 2 | 0 | 5 | 0.800 | 1.000 | 0.600 | 1.000 | 0.750 | 0.920 |
| Liver | Metabolomics | 96 | 10 (5/5) | 5 | 0 | 1 | 4 | 0.900 | 0.833 | 1.000 | 0.800 | 0.909 | 0.960 |
| Liver | Metabolomics | 120 | 10 (5/5) | 4 | 1 | 0 | 5 | 0.900 | 1.000 | 0.800 | 1.000 | 0.889 | 1.000 |
| Liver | Metabolomics | 144 | 10 (5/5) | 5 | 0 | 1 | 4 | 0.900 | 0.833 | 1.000 | 0.800 | 0.909 | 1.000 |
| Liver | Metabolomics | **168** | 10 (5/5) | 5 | 0 | 0 | 5 | 1.000 | 1.000 | 1.000 | 1.000 | 1.000 | 1.000 |
| Liver | Microbial profiling | **0** | 10 (5/5) | 4 | 1 | 4 | 1 | 0.500 | 0.500 | 0.800 | 0.200 | 0.615 | 0.600 |
| Liver | Microbial profiling | 12 | 10 (5/5) | 5 | 0 | 3 | 2 | 0.700 | 0.625 | 1.000 | 0.400 | 0.769 | 0.680 |
| Liver | Microbial profiling | 24 | 10 (5/5) | 4 | 1 | 0 | 5 | 0.900 | 1.000 | 0.800 | 1.000 | 0.889 | 0.880 |
| Liver | Microbial profiling | 48 | 10 (5/5) | 5 | 0 | 0 | 5 | 1.000 | 1.000 | 1.000 | 1.000 | 1.000 | 1.000 |
| Liver | Microbial profiling | **72** | 10 (5/5) | 5 | 0 | 0 | 5 | 1.000 | 1.000 | 1.000 | 1.000 | 1.000 | 1.000 |
| Liver | Microbial profiling | 96 | 10 (5/5) | 5 | 0 | 0 | 5 | 1.000 | 1.000 | 1.000 | 1.000 | 1.000 | 1.000 |
| Liver | Microbial profiling | 120 | 10 (5/5) | 1 | 4 | 1 | 4 | 0.500 | 0.500 | 0.200 | 0.800 | 0.286 | 0.760 |
| Liver | Microbial profiling | 144 | 10 (5/5) | 4 | 1 | 0 | 5 | 0.900 | 1.000 | 0.800 | 1.000 | 0.889 | 1.000 |
| Liver | Microbial profiling | **168** | 10 (5/5) | 5 | 0 | 0 | 5 | 1.000 | 1.000 | 1.000 | 1.000 | 1.000 | 1.000 |
| Liver | Integrated | **0** | 10 (5/5) | 5 | 0 | 0 | 5 | 1.000 | 1.000 | 1.000 | 1.000 | 1.000 | 1.000 |
| Liver | Integrated | 12 | 10 (5/5) | 5 | 0 | 1 | 4 | 0.900 | 0.833 | 1.000 | 0.800 | 0.909 | 1.000 |
| Liver | Integrated | 24 | 10 (5/5) | 5 | 0 | 0 | 5 | 1.000 | 1.000 | 1.000 | 1.000 | 1.000 | 1.000 |
| Liver | Integrated | 48 | 10 (5/5) | 5 | 0 | 0 | 5 | 1.000 | 1.000 | 1.000 | 1.000 | 1.000 | 1.000 |
| Liver | Integrated | **72** | 10 (5/5) | 4 | 1 | 0 | 5 | 0.900 | 1.000 | 0.800 | 1.000 | 0.889 | 1.000 |
| Liver | Integrated | 96 | 10 (5/5) | 5 | 0 | 1 | 4 | 0.900 | 0.833 | 1.000 | 0.800 | 0.909 | 1.000 |
| Liver | Integrated | 120 | 10 (5/5) | 3 | 2 | 0 | 5 | 0.800 | 1.000 | 0.600 | 1.000 | 0.750 | 1.000 |
| Liver | Integrated | 144 | 10 (5/5) | 5 | 0 | 0 | 5 | 1.000 | 1.000 | 1.000 | 1.000 | 1.000 | 1.000 |
| Liver | Integrated | **168** | 10 (5/5) | 5 | 0 | 0 | 5 | 1.000 | 1.000 | 1.000 | 1.000 | 1.000 | 1.000 |
| Kidney | Metabolomics | **0** | 10 (5/5) | 5 | 0 | 0 | 5 | 1.000 | 1.000 | 1.000 | 1.000 | 1.000 | 1.000 |
| Kidney | Metabolomics | 12 | 10 (5/5) | 5 | 0 | 0 | 5 | 1.000 | 1.000 | 1.000 | 1.000 | 1.000 | 1.000 |
| Kidney | Metabolomics | 24 | 10 (5/5) | 5 | 0 | 0 | 5 | 1.000 | 1.000 | 1.000 | 1.000 | 1.000 | 1.000 |
| Kidney | Metabolomics | 48 | 10 (5/5) | 4 | 1 | 0 | 5 | 0.900 | 1.000 | 0.800 | 1.000 | 0.889 | 1.000 |
| Kidney | Metabolomics | **72** | 10 (5/5) | 5 | 0 | 0 | 5 | 1.000 | 1.000 | 1.000 | 1.000 | 1.000 | 1.000 |
| Kidney | Metabolomics | 96 | 10 (5/5) | 5 | 0 | 0 | 5 | 1.000 | 1.000 | 1.000 | 1.000 | 1.000 | 1.000 |
| Kidney | Metabolomics | 120 | 10 (5/5) | 4 | 1 | 1 | 4 | 0.800 | 0.800 | 0.800 | 0.800 | 0.800 | 0.960 |
| Kidney | Metabolomics | 144 | 10 (5/5) | 5 | 0 | 0 | 5 | 1.000 | 1.000 | 1.000 | 1.000 | 1.000 | 1.000 |
| Kidney | Metabolomics | **168** | 10 (5/5) | 5 | 0 | 0 | 5 | 1.000 | 1.000 | 1.000 | 1.000 | 1.000 | 1.000 |
| Kidney | Microbial profiling | **0** | 10 (5/5) | 4 | 1 | 4 | 1 | 0.500 | 0.500 | 0.800 | 0.200 | 0.615 | 0.400 |
| Kidney | Microbial profiling | 12 | 10 (5/5) | 3 | 2 | 2 | 3 | 0.600 | 0.600 | 0.600 | 0.600 | 0.600 | 0.520 |
| Kidney | Microbial profiling | 24 | 10 (5/5) | 4 | 1 | 2 | 3 | 0.700 | 0.667 | 0.800 | 0.600 | 0.727 | 0.840 |
| Kidney | Microbial profiling | 48 | 10 (5/5) | 4 | 1 | 0 | 5 | 0.900 | 1.000 | 0.800 | 1.000 | 0.889 | 1.000 |
| Kidney | Microbial profiling | **72** | 10 (5/5) | 5 | 0 | 1 | 4 | 0.900 | 0.833 | 1.000 | 0.800 | 0.909 | 1.000 |
| Kidney | Microbial profiling | 96 | 10 (5/5) | 5 | 0 | 0 | 5 | 1.000 | 1.000 | 1.000 | 1.000 | 1.000 | 1.000 |
| Kidney | Microbial profiling | 120 | 10 (5/5) | 3 | 2 | 1 | 4 | 0.700 | 0.750 | 0.600 | 0.800 | 0.667 | 0.800 |
| Kidney | Microbial profiling | 144 | 10 (5/5) | 3 | 2 | 0 | 5 | 0.800 | 1.000 | 0.600 | 1.000 | 0.750 | 1.000 |
| Kidney | Microbial profiling | **168** | 10 (5/5) | 5 | 0 | 1 | 4 | 0.900 | 0.833 | 1.000 | 0.800 | 0.909 | 1.000 |
| Kidney | Integrated | **0** | 10 (5/5) | 5 | 0 | 0 | 5 | 1.000 | 1.000 | 1.000 | 1.000 | 1.000 | 1.000 |
| Kidney | Integrated | 12 | 10 (5/5) | 5 | 0 | 0 | 5 | 1.000 | 1.000 | 1.000 | 1.000 | 1.000 | 1.000 |
| Kidney | Integrated | 24 | 10 (5/5) | 5 | 0 | 0 | 5 | 1.000 | 1.000 | 1.000 | 1.000 | 1.000 | 1.000 |
| Kidney | Integrated | 48 | 10 (5/5) | 4 | 1 | 0 | 5 | 0.900 | 1.000 | 0.800 | 1.000 | 0.889 | 1.000 |
| Kidney | Integrated | **72** | 10 (5/5) | 5 | 0 | 0 | 5 | 1.000 | 1.000 | 1.000 | 1.000 | 1.000 | 1.000 |
| Kidney | Integrated | 96 | 10 (5/5) | 5 | 0 | 0 | 5 | 1.000 | 1.000 | 1.000 | 1.000 | 1.000 | 1.000 |
| Kidney | Integrated | 120 | 10 (5/5) | 4 | 1 | 0 | 5 | 0.900 | 1.000 | 0.800 | 1.000 | 0.889 | 0.960 |
| Kidney | Integrated | 144 | 10 (5/5) | 5 | 0 | 0 | 5 | 1.000 | 1.000 | 1.000 | 1.000 | 1.000 | 1.000 |
| Kidney | Integrated | **168** | 10 (5/5) | 5 | 0 | 0 | 5 | 1.000 | 1.000 | 1.000 | 1.000 | 1.000 | 1.000 |
| Skeletal muscle | Metabolomics | **0** | 10 (5/5) | 4 | 1 | 0 | 5 | 0.900 | 1.000 | 0.800 | 1.000 | 0.889 | 1.000 |
| Skeletal muscle | Metabolomics | 12 | 10 (5/5) | 5 | 0 | 0 | 5 | 1.000 | 1.000 | 1.000 | 1.000 | 1.000 | 1.000 |
| Skeletal muscle | Metabolomics | 24 | 10 (5/5) | 5 | 0 | 0 | 5 | 1.000 | 1.000 | 1.000 | 1.000 | 1.000 | 1.000 |
| Skeletal muscle | Metabolomics | 48 | 10 (5/5) | 5 | 0 | 0 | 5 | 1.000 | 1.000 | 1.000 | 1.000 | 1.000 | 1.000 |
| Skeletal muscle | Metabolomics | **72** | 10 (5/5) | 4 | 1 | 0 | 5 | 0.900 | 1.000 | 0.800 | 1.000 | 0.889 | 0.840 |
| Skeletal muscle | Metabolomics | 96 | 10 (5/5) | 5 | 0 | 2 | 3 | 0.800 | 0.714 | 1.000 | 0.600 | 0.833 | 1.000 |
| Skeletal muscle | Metabolomics | 120 | 10 (5/5) | 3 | 2 | 0 | 5 | 0.800 | 1.000 | 0.600 | 1.000 | 0.750 | 0.800 |
| Skeletal muscle | Metabolomics | 144 | 10 (5/5) | 5 | 0 | 1 | 4 | 0.900 | 0.833 | 1.000 | 0.800 | 0.909 | 1.000 |
| Skeletal muscle | Metabolomics | **168** | 10 (5/5) | 5 | 0 | 0 | 5 | 1.000 | 1.000 | 1.000 | 1.000 | 1.000 | 1.000 |
| Skeletal muscle | Microbial profiling | **0** | 10 (5/5) | 5 | 0 | 2 | 3 | 0.800 | 0.714 | 1.000 | 0.600 | 0.833 | 1.000 |
| Skeletal muscle | Microbial profiling | 12 | 10 (5/5) | 5 | 0 | 2 | 3 | 0.800 | 0.714 | 1.000 | 0.600 | 0.833 | 0.840 |
| Skeletal muscle | Microbial profiling | 24 | 10 (5/5) | 4 | 1 | 1 | 4 | 0.800 | 0.800 | 0.800 | 0.800 | 0.800 | 0.760 |
| Skeletal muscle | Microbial profiling | 48 | 10 (5/5) | 5 | 0 | 0 | 5 | 1.000 | 1.000 | 1.000 | 1.000 | 1.000 | 1.000 |
| Skeletal muscle | Microbial profiling | **72** | 10 (5/5) | 5 | 0 | 1 | 4 | 0.900 | 0.833 | 1.000 | 0.800 | 0.909 | 1.000 |
| Skeletal muscle | Microbial profiling | 96 | 10 (5/5) | 4 | 1 | 1 | 4 | 0.800 | 0.800 | 0.800 | 0.800 | 0.800 | 0.960 |
| Skeletal muscle | Microbial profiling | 120 | 10 (5/5) | 1 | 4 | 1 | 4 | 0.500 | 0.500 | 0.200 | 0.800 | 0.286 | 0.800 |
| Skeletal muscle | Microbial profiling | 144 | 10 (5/5) | 4 | 1 | 1 | 4 | 0.800 | 0.800 | 0.800 | 0.800 | 0.800 | 0.960 |
| Skeletal muscle | Microbial profiling | **168** | 10 (5/5) | 5 | 0 | 0 | 5 | 1.000 | 1.000 | 1.000 | 1.000 | 1.000 | 1.000 |
| Skeletal muscle | Integrated | **0** | 10 (5/5) | 5 | 0 | 0 | 5 | 1.000 | 1.000 | 1.000 | 1.000 | 1.000 | 1.000 |
| Skeletal muscle | Integrated | 12 | 10 (5/5) | 5 | 0 | 0 | 5 | 1.000 | 1.000 | 1.000 | 1.000 | 1.000 | 1.000 |
| Skeletal muscle | Integrated | 24 | 10 (5/5) | 5 | 0 | 0 | 5 | 1.000 | 1.000 | 1.000 | 1.000 | 1.000 | 1.000 |
| Skeletal muscle | Integrated | 48 | 10 (5/5) | 5 | 0 | 0 | 5 | 1.000 | 1.000 | 1.000 | 1.000 | 1.000 | 1.000 |
| Skeletal muscle | Integrated | **72** | 10 (5/5) | 4 | 1 | 0 | 5 | 0.900 | 1.000 | 0.800 | 1.000 | 0.889 | 0.960 |
| Skeletal muscle | Integrated | 96 | 10 (5/5) | 5 | 0 | 2 | 3 | 0.800 | 0.714 | 1.000 | 0.600 | 0.833 | 1.000 |
| Skeletal muscle | Integrated | 120 | 10 (5/5) | 4 | 1 | 0 | 5 | 0.900 | 1.000 | 0.800 | 1.000 | 0.889 | 0.840 |
| Skeletal muscle | Integrated | 144 | 10 (5/5) | 5 | 0 | 0 | 5 | 1.000 | 1.000 | 1.000 | 1.000 | 1.000 | 1.000 |
| Skeletal muscle | Integrated | **168** | 10 (5/5) | 5 | 0 | 0 | 5 | 1.000 | 1.000 | 1.000 | 1.000 | 1.000 | 1.000 |

Note: Aggregated OOF probabilities were calculated at the sample level by averaging the five repeat-specific OOF probabilities obtained for each sample during repeated five-fold cross-validation. Metrics were then calculated separately within each available PMI stratum using a predicted-probability threshold of 0.5, with HS treated as the positive class. Most development-cohort PMI strata contained 5 HS and 5 C samples; the brain at 72 h contained 4 HS and 4 C samples. PMI points not available for a given organ were omitted. Integrated indicates models using 10 selected metabolic features and 10 selected genus-level microbial features. HS, heatstroke death group; C, control group; PMI, postmortem interval; OOF, out-of-fold; TP, true positive; FN, false negative; FP, false positive; TN, true negative; AUC, area under the receiver operating characteristic curve.
