# Supplementary material for: Comparative evaluation of metabolomic, microbial, and integrated features for distinguishing a rat model of heatstroke death from cervical-dislocation controls and estimating postmortem interval across organs
Source: Front Microbiol. 2026 Sep 29;17:1897987. doi: 10.3389/fmicb.2026.1897987 (PMC13658517; doi:10.3389/fmicb.2026.1897987)
Supplement: Supplementary file 3 [file Table_8.DOCX]

**Supplementary Table S8. PMI-stratified external-validation performance of HS/C classification models across organs and feature strategies.**

| **Organ** | **Feature strategy** | **PMI (h)** | **n (HS/C)** | **TP** | **FN** | **FP** | **TN** | **Accuracy** | **Precision** | **Sensitivity** | **Specificity** | **F1-score** | **AUC** |
| --- | --- | --- | --- | --- | --- | --- | --- | --- | --- | --- | --- | --- | --- |
| Brain | Metabolomics | **0** | 6 (3/3) | 3 | 0 | 0 | 3 | 1.000 | 1.000 | 1.000 | 1.000 | 1.000 | 1.000 |
| Brain | Metabolomics | 12 | 6 (3/3) | 3 | 0 | 0 | 3 | 1.000 | 1.000 | 1.000 | 1.000 | 1.000 | 1.000 |
| Brain | Metabolomics | 24 | 6 (3/3) | 3 | 0 | 0 | 3 | 1.000 | 1.000 | 1.000 | 1.000 | 1.000 | 1.000 |
| Brain | Metabolomics | 48 | 6 (3/3) | 3 | 0 | 0 | 3 | 1.000 | 1.000 | 1.000 | 1.000 | 1.000 | 1.000 |
| Brain | Metabolomics | **72** | 6 (3/3) | 3 | 0 | 0 | 3 | 1.000 | 1.000 | 1.000 | 1.000 | 1.000 | 1.000 |
| Brain | Microbial profiling | **0** | 6 (3/3) | 3 | 0 | 0 | 3 | 1.000 | 1.000 | 1.000 | 1.000 | 1.000 | 1.000 |
| Brain | Microbial profiling | 12 | 6 (3/3) | 3 | 0 | 0 | 3 | 1.000 | 1.000 | 1.000 | 1.000 | 1.000 | 1.000 |
| Brain | Microbial profiling | 24 | 6 (3/3) | 3 | 0 | 2 | 1 | 0.667 | 0.600 | 1.000 | 0.333 | 0.750 | 1.000 |
| Brain | Microbial profiling | 48 | 6 (3/3) | 3 | 0 | 0 | 3 | 1.000 | 1.000 | 1.000 | 1.000 | 1.000 | 1.000 |
| Brain | Microbial profiling | **72** | 6 (3/3) | 3 | 0 | 1 | 2 | 0.833 | 0.750 | 1.000 | 0.667 | 0.857 | 0.778 |
| Brain | Integrated | **0** | 6 (3/3) | 3 | 0 | 0 | 3 | 1.000 | 1.000 | 1.000 | 1.000 | 1.000 | 1.000 |
| Brain | Integrated | 12 | 6 (3/3) | 3 | 0 | 0 | 3 | 1.000 | 1.000 | 1.000 | 1.000 | 1.000 | 1.000 |
| Brain | Integrated | 24 | 6 (3/3) | 3 | 0 | 0 | 3 | 1.000 | 1.000 | 1.000 | 1.000 | 1.000 | 1.000 |
| Brain | Integrated | 48 | 6 (3/3) | 3 | 0 | 0 | 3 | 1.000 | 1.000 | 1.000 | 1.000 | 1.000 | 1.000 |
| Brain | Integrated | **72** | 6 (3/3) | 3 | 0 | 0 | 3 | 1.000 | 1.000 | 1.000 | 1.000 | 1.000 | 1.000 |
| Cerebellum | Metabolomics | **0** | 6 (3/3) | 3 | 0 | 0 | 3 | 1.000 | 1.000 | 1.000 | 1.000 | 1.000 | 1.000 |
| Cerebellum | Metabolomics | 12 | 6 (3/3) | 3 | 0 | 0 | 3 | 1.000 | 1.000 | 1.000 | 1.000 | 1.000 | 1.000 |
| Cerebellum | Metabolomics | 24 | 6 (3/3) | 3 | 0 | 0 | 3 | 1.000 | 1.000 | 1.000 | 1.000 | 1.000 | 1.000 |
| Cerebellum | Metabolomics | 48 | 6 (3/3) | 3 | 0 | 0 | 3 | 1.000 | 1.000 | 1.000 | 1.000 | 1.000 | 1.000 |
| Cerebellum | Microbial profiling | **0** | 6 (3/3) | 3 | 0 | 0 | 3 | 1.000 | 1.000 | 1.000 | 1.000 | 1.000 | 1.000 |
| Cerebellum | Microbial profiling | 12 | 6 (3/3) | 3 | 0 | 0 | 3 | 1.000 | 1.000 | 1.000 | 1.000 | 1.000 | 1.000 |
| Cerebellum | Microbial profiling | 24 | 6 (3/3) | 3 | 0 | 0 | 3 | 1.000 | 1.000 | 1.000 | 1.000 | 1.000 | 1.000 |
| Cerebellum | Microbial profiling | 48 | 6 (3/3) | 3 | 0 | 0 | 3 | 1.000 | 1.000 | 1.000 | 1.000 | 1.000 | 1.000 |
| Cerebellum | Integrated | **0** | 6 (3/3) | 3 | 0 | 0 | 3 | 1.000 | 1.000 | 1.000 | 1.000 | 1.000 | 1.000 |
| Cerebellum | Integrated | 12 | 6 (3/3) | 3 | 0 | 0 | 3 | 1.000 | 1.000 | 1.000 | 1.000 | 1.000 | 1.000 |
| Cerebellum | Integrated | 24 | 6 (3/3) | 3 | 0 | 0 | 3 | 1.000 | 1.000 | 1.000 | 1.000 | 1.000 | 1.000 |
| Cerebellum | Integrated | 48 | 6 (3/3) | 3 | 0 | 0 | 3 | 1.000 | 1.000 | 1.000 | 1.000 | 1.000 | 1.000 |
| Liver | Metabolomics | **0** | 6 (3/3) | 3 | 0 | 0 | 3 | 1.000 | 1.000 | 1.000 | 1.000 | 1.000 | 1.000 |
| Liver | Metabolomics | 12 | 6 (3/3) | 3 | 0 | 0 | 3 | 1.000 | 1.000 | 1.000 | 1.000 | 1.000 | 1.000 |
| Liver | Metabolomics | 24 | 6 (3/3) | 3 | 0 | 0 | 3 | 1.000 | 1.000 | 1.000 | 1.000 | 1.000 | 1.000 |
| Liver | Metabolomics | 48 | 6 (3/3) | 3 | 0 | 0 | 3 | 1.000 | 1.000 | 1.000 | 1.000 | 1.000 | 1.000 |
| Liver | Metabolomics | **72** | 6 (3/3) | 3 | 0 | 0 | 3 | 1.000 | 1.000 | 1.000 | 1.000 | 1.000 | 1.000 |
| Liver | Metabolomics | 96 | 6 (3/3) | 3 | 0 | 0 | 3 | 1.000 | 1.000 | 1.000 | 1.000 | 1.000 | 1.000 |
| Liver | Metabolomics | 120 | 6 (3/3) | 3 | 0 | 0 | 3 | 1.000 | 1.000 | 1.000 | 1.000 | 1.000 | 1.000 |
| Liver | Metabolomics | 144 | 6 (3/3) | 3 | 0 | 0 | 3 | 1.000 | 1.000 | 1.000 | 1.000 | 1.000 | 1.000 |
| Liver | Metabolomics | **168** | 6 (3/3) | 3 | 0 | 0 | 3 | 1.000 | 1.000 | 1.000 | 1.000 | 1.000 | 1.000 |
| Liver | Microbial profiling | **0** | 6 (3/3) | 3 | 0 | 0 | 3 | 1.000 | 1.000 | 1.000 | 1.000 | 1.000 | 1.000 |
| Liver | Microbial profiling | 12 | 6 (3/3) | 3 | 0 | 0 | 3 | 1.000 | 1.000 | 1.000 | 1.000 | 1.000 | 1.000 |
| Liver | Microbial profiling | 24 | 6 (3/3) | 2 | 1 | 0 | 3 | 0.833 | 1.000 | 0.667 | 1.000 | 0.800 | 1.000 |
| Liver | Microbial profiling | 48 | 6 (3/3) | 3 | 0 | 0 | 3 | 1.000 | 1.000 | 1.000 | 1.000 | 1.000 | 1.000 |
| Liver | Microbial profiling | **72** | 6 (3/3) | 3 | 0 | 0 | 3 | 1.000 | 1.000 | 1.000 | 1.000 | 1.000 | 1.000 |
| Liver | Microbial profiling | 96 | 6 (3/3) | 3 | 0 | 0 | 3 | 1.000 | 1.000 | 1.000 | 1.000 | 1.000 | 1.000 |
| Liver | Microbial profiling | 120 | 6 (3/3) | 0 | 3 | 0 | 3 | 0.500 | 0.000 | 0.000 | 1.000 | 0.000 | 1.000 |
| Liver | Microbial profiling | 144 | 6 (3/3) | 3 | 0 | 0 | 3 | 1.000 | 1.000 | 1.000 | 1.000 | 1.000 | 1.000 |
| Liver | Microbial profiling | **168** | 6 (3/3) | 3 | 0 | 0 | 3 | 1.000 | 1.000 | 1.000 | 1.000 | 1.000 | 1.000 |
| Liver | Integrated | **0** | 6 (3/3) | 3 | 0 | 0 | 3 | 1.000 | 1.000 | 1.000 | 1.000 | 1.000 | 1.000 |
| Liver | Integrated | 12 | 6 (3/3) | 3 | 0 | 0 | 3 | 1.000 | 1.000 | 1.000 | 1.000 | 1.000 | 1.000 |
| Liver | Integrated | 24 | 6 (3/3) | 3 | 0 | 0 | 3 | 1.000 | 1.000 | 1.000 | 1.000 | 1.000 | 1.000 |
| Liver | Integrated | 48 | 6 (3/3) | 3 | 0 | 0 | 3 | 1.000 | 1.000 | 1.000 | 1.000 | 1.000 | 1.000 |
| Liver | Integrated | **72** | 6 (3/3) | 3 | 0 | 0 | 3 | 1.000 | 1.000 | 1.000 | 1.000 | 1.000 | 1.000 |
| Liver | Integrated | 96 | 6 (3/3) | 3 | 0 | 0 | 3 | 1.000 | 1.000 | 1.000 | 1.000 | 1.000 | 1.000 |
| Liver | Integrated | 120 | 6 (3/3) | 3 | 0 | 0 | 3 | 1.000 | 1.000 | 1.000 | 1.000 | 1.000 | 1.000 |
| Liver | Integrated | 144 | 6 (3/3) | 3 | 0 | 0 | 3 | 1.000 | 1.000 | 1.000 | 1.000 | 1.000 | 1.000 |
| Liver | Integrated | **168** | 6 (3/3) | 3 | 0 | 0 | 3 | 1.000 | 1.000 | 1.000 | 1.000 | 1.000 | 1.000 |
| Kidney | Metabolomics | **0** | 6 (3/3) | 3 | 0 | 0 | 3 | 1.000 | 1.000 | 1.000 | 1.000 | 1.000 | 1.000 |
| Kidney | Metabolomics | 12 | 6 (3/3) | 3 | 0 | 0 | 3 | 1.000 | 1.000 | 1.000 | 1.000 | 1.000 | 1.000 |
| Kidney | Metabolomics | 24 | 6 (3/3) | 3 | 0 | 0 | 3 | 1.000 | 1.000 | 1.000 | 1.000 | 1.000 | 1.000 |
| Kidney | Metabolomics | 48 | 6 (3/3) | 3 | 0 | 0 | 3 | 1.000 | 1.000 | 1.000 | 1.000 | 1.000 | 1.000 |
| Kidney | Metabolomics | **72** | 6 (3/3) | 3 | 0 | 0 | 3 | 1.000 | 1.000 | 1.000 | 1.000 | 1.000 | 1.000 |
| Kidney | Metabolomics | 96 | 6 (3/3) | 3 | 0 | 0 | 3 | 1.000 | 1.000 | 1.000 | 1.000 | 1.000 | 1.000 |
| Kidney | Metabolomics | 120 | 6 (3/3) | 3 | 0 | 0 | 3 | 1.000 | 1.000 | 1.000 | 1.000 | 1.000 | 1.000 |
| Kidney | Metabolomics | 144 | 6 (3/3) | 3 | 0 | 0 | 3 | 1.000 | 1.000 | 1.000 | 1.000 | 1.000 | 1.000 |
| Kidney | Metabolomics | **168** | 6 (3/3) | 3 | 0 | 0 | 3 | 1.000 | 1.000 | 1.000 | 1.000 | 1.000 | 1.000 |
| Kidney | Microbial profiling | **0** | 6 (3/3) | 1 | 2 | 2 | 1 | 0.333 | 0.333 | 0.333 | 0.333 | 0.333 | 0.222 |
| Kidney | Microbial profiling | 12 | 6 (3/3) | 3 | 0 | 0 | 3 | 1.000 | 1.000 | 1.000 | 1.000 | 1.000 | 1.000 |
| Kidney | Microbial profiling | 24 | 6 (3/3) | 3 | 0 | 0 | 3 | 1.000 | 1.000 | 1.000 | 1.000 | 1.000 | 1.000 |
| Kidney | Microbial profiling | 48 | 6 (3/3) | 3 | 0 | 0 | 3 | 1.000 | 1.000 | 1.000 | 1.000 | 1.000 | 1.000 |
| Kidney | Microbial profiling | **72** | 6 (3/3) | 3 | 0 | 0 | 3 | 1.000 | 1.000 | 1.000 | 1.000 | 1.000 | 1.000 |
| Kidney | Microbial profiling | 96 | 6 (3/3) | 3 | 0 | 0 | 3 | 1.000 | 1.000 | 1.000 | 1.000 | 1.000 | 1.000 |
| Kidney | Microbial profiling | 120 | 6 (3/3) | 0 | 3 | 0 | 3 | 0.500 | 0.000 | 0.000 | 1.000 | 0.000 | 1.000 |
| Kidney | Microbial profiling | 144 | 6 (3/3) | 3 | 0 | 0 | 3 | 1.000 | 1.000 | 1.000 | 1.000 | 1.000 | 1.000 |
| Kidney | Microbial profiling | **168** | 6 (3/3) | 3 | 0 | 0 | 3 | 1.000 | 1.000 | 1.000 | 1.000 | 1.000 | 1.000 |
| Kidney | Integrated | **0** | 6 (3/3) | 3 | 0 | 0 | 3 | 1.000 | 1.000 | 1.000 | 1.000 | 1.000 | 1.000 |
| Kidney | Integrated | 12 | 6 (3/3) | 3 | 0 | 0 | 3 | 1.000 | 1.000 | 1.000 | 1.000 | 1.000 | 1.000 |
| Kidney | Integrated | 24 | 6 (3/3) | 3 | 0 | 0 | 3 | 1.000 | 1.000 | 1.000 | 1.000 | 1.000 | 1.000 |
| Kidney | Integrated | 48 | 6 (3/3) | 3 | 0 | 0 | 3 | 1.000 | 1.000 | 1.000 | 1.000 | 1.000 | 1.000 |
| Kidney | Integrated | **72** | 6 (3/3) | 3 | 0 | 0 | 3 | 1.000 | 1.000 | 1.000 | 1.000 | 1.000 | 1.000 |
| Kidney | Integrated | 96 | 6 (3/3) | 3 | 0 | 0 | 3 | 1.000 | 1.000 | 1.000 | 1.000 | 1.000 | 1.000 |
| Kidney | Integrated | 120 | 6 (3/3) | 3 | 0 | 0 | 3 | 1.000 | 1.000 | 1.000 | 1.000 | 1.000 | 1.000 |
| Kidney | Integrated | 144 | 6 (3/3) | 3 | 0 | 0 | 3 | 1.000 | 1.000 | 1.000 | 1.000 | 1.000 | 1.000 |
| Kidney | Integrated | **168** | 6 (3/3) | 3 | 0 | 0 | 3 | 1.000 | 1.000 | 1.000 | 1.000 | 1.000 | 1.000 |
| Skeletal muscle | Metabolomics | **0** | 6 (3/3) | 3 | 0 | 0 | 3 | 1.000 | 1.000 | 1.000 | 1.000 | 1.000 | 1.000 |
| Skeletal muscle | Metabolomics | 12 | 6 (3/3) | 3 | 0 | 0 | 3 | 1.000 | 1.000 | 1.000 | 1.000 | 1.000 | 1.000 |
| Skeletal muscle | Metabolomics | 24 | 6 (3/3) | 3 | 0 | 0 | 3 | 1.000 | 1.000 | 1.000 | 1.000 | 1.000 | 1.000 |
| Skeletal muscle | Metabolomics | 48 | 6 (3/3) | 3 | 0 | 0 | 3 | 1.000 | 1.000 | 1.000 | 1.000 | 1.000 | 1.000 |
| Skeletal muscle | Metabolomics | **72** | 6 (3/3) | 3 | 0 | 0 | 3 | 1.000 | 1.000 | 1.000 | 1.000 | 1.000 | 1.000 |
| Skeletal muscle | Metabolomics | 96 | 6 (3/3) | 3 | 0 | 0 | 3 | 1.000 | 1.000 | 1.000 | 1.000 | 1.000 | 1.000 |
| Skeletal muscle | Metabolomics | 120 | 6 (3/3) | 3 | 0 | 0 | 3 | 1.000 | 1.000 | 1.000 | 1.000 | 1.000 | 1.000 |
| Skeletal muscle | Metabolomics | 144 | 6 (3/3) | 3 | 0 | 0 | 3 | 1.000 | 1.000 | 1.000 | 1.000 | 1.000 | 1.000 |
| Skeletal muscle | Metabolomics | **168** | 6 (3/3) | 3 | 0 | 0 | 3 | 1.000 | 1.000 | 1.000 | 1.000 | 1.000 | 1.000 |
| Skeletal muscle | Microbial profiling | **0** | 6 (3/3) | 3 | 0 | 0 | 3 | 1.000 | 1.000 | 1.000 | 1.000 | 1.000 | 1.000 |
| Skeletal muscle | Microbial profiling | 12 | 6 (3/3) | 3 | 0 | 0 | 3 | 1.000 | 1.000 | 1.000 | 1.000 | 1.000 | 1.000 |
| Skeletal muscle | Microbial profiling | 24 | 6 (3/3) | 3 | 0 | 0 | 3 | 1.000 | 1.000 | 1.000 | 1.000 | 1.000 | 1.000 |
| Skeletal muscle | Microbial profiling | 48 | 6 (3/3) | 3 | 0 | 0 | 3 | 1.000 | 1.000 | 1.000 | 1.000 | 1.000 | 1.000 |
| Skeletal muscle | Microbial profiling | **72** | 6 (3/3) | 3 | 0 | 0 | 3 | 1.000 | 1.000 | 1.000 | 1.000 | 1.000 | 1.000 |
| Skeletal muscle | Microbial profiling | 96 | 6 (3/3) | 2 | 1 | 0 | 3 | 0.833 | 1.000 | 0.667 | 1.000 | 0.800 | 1.000 |
| Skeletal muscle | Microbial profiling | 120 | 6 (3/3) | 2 | 1 | 0 | 3 | 0.833 | 1.000 | 0.667 | 1.000 | 0.800 | 1.000 |
| Skeletal muscle | Microbial profiling | 144 | 6 (3/3) | 3 | 0 | 0 | 3 | 1.000 | 1.000 | 1.000 | 1.000 | 1.000 | 1.000 |
| Skeletal muscle | Microbial profiling | **168** | 6 (3/3) | 3 | 0 | 0 | 3 | 1.000 | 1.000 | 1.000 | 1.000 | 1.000 | 1.000 |
| Skeletal muscle | Integrated | **0** | 6 (3/3) | 3 | 0 | 0 | 3 | 1.000 | 1.000 | 1.000 | 1.000 | 1.000 | 1.000 |
| Skeletal muscle | Integrated | 12 | 6 (3/3) | 3 | 0 | 0 | 3 | 1.000 | 1.000 | 1.000 | 1.000 | 1.000 | 1.000 |
| Skeletal muscle | Integrated | 24 | 6 (3/3) | 3 | 0 | 0 | 3 | 1.000 | 1.000 | 1.000 | 1.000 | 1.000 | 1.000 |
| Skeletal muscle | Integrated | 48 | 6 (3/3) | 3 | 0 | 0 | 3 | 1.000 | 1.000 | 1.000 | 1.000 | 1.000 | 1.000 |
| Skeletal muscle | Integrated | **72** | 6 (3/3) | 3 | 0 | 0 | 3 | 1.000 | 1.000 | 1.000 | 1.000 | 1.000 | 1.000 |
| Skeletal muscle | Integrated | 96 | 6 (3/3) | 3 | 0 | 0 | 3 | 1.000 | 1.000 | 1.000 | 1.000 | 1.000 | 1.000 |
| Skeletal muscle | Integrated | 120 | 6 (3/3) | 3 | 0 | 0 | 3 | 1.000 | 1.000 | 1.000 | 1.000 | 1.000 | 1.000 |
| Skeletal muscle | Integrated | 144 | 6 (3/3) | 3 | 0 | 0 | 3 | 1.000 | 1.000 | 1.000 | 1.000 | 1.000 | 1.000 |
| Skeletal muscle | Integrated | **168** | 6 (3/3) | 3 | 0 | 0 | 3 | 1.000 | 1.000 | 1.000 | 1.000 | 1.000 | 1.000 |

Note: External validation was performed using independent organ-specific HS/C samples matched by PMI. Metrics were calculated separately within each available PMI stratum using a predicted-probability threshold of 0.5, with HS treated as the positive class. Each reported PMI stratum contained 3 HS and 3 C samples. Cerebellum analyses were restricted to the matched PMI points of 0, 12, 24, and 48 h. Because each PMI-specific external-validation subset contained only six samples, these results should be interpreted descriptively. Integrated indicates models using 10 selected metabolic features and 10 selected genus-level microbial features. HS, heatstroke death group; C, control group; PMI, postmortem interval; TP, true positive; FN, false negative; FP, false positive; TN, true negative; AUC, area under the receiver operating characteristic curve.
